# Cipaglucosidase alfa plus miglustat: linking mechanism of action to clinical outcomes in late-onset Pompe disease

**DOI:** 10.3389/fneur.2024.1451512

**Published:** 2024-10-18

**Authors:** Barry J. Byrne, Giancarlo Parenti, Benedikt Schoser, Ans T. van der Ploeg, Hung Do, Brian Fox, Mitchell Goldman, Franklin K. Johnson, Jia Kang, Nickita Mehta, John Mondick, M. Osman Sheikh, Sheela Sitaraman Das, Steven Tuske, Jon Brudvig, Jill M. Weimer, Tahseen Mozaffar

**Affiliations:** ^1^Department of Pediatrics in the College of Medicine, University of Florida, Gainesville, FL, United States; ^2^Metabolic Unit, Department of Translational Medical Sciences, University of Naples Federico II, Naples, Italy; ^3^Telethon Institute of Genetics and Medicine, Pozzuoli, Italy; ^4^Friedrich-Baur-Institute, Department of Neurology, LMU University Hospital, LMU Munich, Munich, Germany; ^5^Erasmus MC University Medical Center, Rotterdam, Netherlands; ^6^M6P-Therapeutics, St Louis, MO, United States; ^7^Amicus Therapeutics, Inc., Princeton, NJ, United States; ^8^Metrum Research Group, Tariffville, CT, United States; ^9^Incyte Corporation, Wilmington, DE, United States; ^10^Department of Neurology, University of California, Irvine, Irvine, CA, United States

**Keywords:** Pompe disease, glycogen storage disease type II, lysosomal storage disorders, enzyme replacement therapy, *n*-butyldeoxynojirimycin

## Abstract

Enzyme replacement therapy (ERT) is the only approved disease-modifying treatment modality for Pompe disease, a rare, inherited metabolic disorder caused by a deficiency in the acid *α*-glucosidase (GAA) enzyme that catabolizes lysosomal glycogen. First-generation recombinant human GAA (rhGAA) ERT (alglucosidase alfa) can slow the progressive muscle degeneration characteristic of the disease. Still, most patients experience diminished efficacy over time, possibly because of poor uptake into target tissues. Next-generation ERTs aim to address this problem by increasing bis-phosphorylated high mannose (bis-M6P) *N*-glycans on rhGAA as these moieties have sufficiently high receptor binding affinity at the resultant low interstitial enzyme concentrations after dosing to drive uptake by the cation-independent mannose 6-phosphate receptor on target cells. However, some approaches introduce bis-M6P onto rhGAA via non-natural linkages that cannot be hydrolyzed by natural human enzymes and thus inhibit the endolysosomal glycan trimming necessary for complete enzyme activation after cell uptake. Furthermore, all rhGAA ERTs face potential inactivation during intravenous delivery (and subsequent non-productive clearance) as GAA is an acid hydrolase that is rapidly denatured in the near-neutral pH of the blood. One new therapy, cipaglucosidase alfa plus miglustat, is hypothesized to address these challenges by combining an enzyme enriched with naturally occurring bis-M6P *N*-glycans with a small-molecule stabilizer. Here, we investigate this hypothesis by analyzing published and new data related to the mechanism of action of the enzyme and stabilizer molecule. Based on an extensive collection of *in vitro*, preclinical, and clinical data, we conclude that cipaglucosidase alfa plus miglustat successfully addresses each of these challenges to offer meaningful advantages in terms of pharmacokinetic exposure, target-cell uptake, endolysosomal processing, and clinical benefit.

## 1 Introduction

Pompe disease is a rare, inherited metabolic disorder caused by biallelic pathogenic variants in the acid *α*-glucosidase (*GAA*) gene, which encodes a lysosomal enzyme that hydrolyzes glycogen (1). When GAA activity is deficient, glycogen accumulates in lysosomes throughout the body, most prominently affecting the heart, skeletal muscle, and smooth muscle (2, 3). This leads to a cascade of cellular dysfunction in processes ranging from lysosomal function to autophagy and endocytic trafficking, culminating in progressive muscle weakness and degeneration (4–6).

Depending on the level of residual enzyme activity, Pompe disease can present as either infantile-onset (IOPD) or late-onset (LOPD) forms. IOPD is the most severe form, resulting from complete or nearly complete loss of GAA activity. IOPD generally presents within the first year of life and is characterized by rapidly progressive hypotonia and myopathy, respiratory insufficiency, and hypertrophic cardiomyopathy (7, 8). Due to the severity of IOPD, Pompe disease is included in newborn screening panels in many countries, including 37 US states and territories (9). Without treatment, IOPD has high mortality within the first year of life (7, 8). LOPD presents later in life as a slowly progressive myopathy. While LOPD does not typically present with severe cardiac involvement, the progressive myopathy leads to respiratory insufficiency, resulting in substantial morbidity and early mortality (1, 7).

Enzyme replacement therapy (ERT) with recombinant human GAA (rhGAA) is the only approved disease-modifying treatment modality for Pompe disease. Alglucosidase alfa (Lumizyme®/Myozyme®; Sanofi) was the first ERT developed for Pompe disease. While the drug improves survival and initially slows disease progression in LOPD, efficacy wanes over 3–5 years of treatment, with many patients continuing to experience deficits in respiratory and muscle function (10–16).

In response to these limitations, one next-generation Pompe ERT, avalglucosidase alfa (Nexviazyme®/Nexviadyme®; Sanofi) was developed using synthetic glycan chemistry to enhance cellular uptake (17–21). Another next-generation therapy, cipaglucosidase alfa plus miglustat (ATB200 + AT2221; Pombiliti® + Opfolda®; Amicus Therapeutics), was developed using naturally enriched bis-phosphorylated high mannose (bis-M6P) *N*-glycans and a small-molecule stabilizer to enhance both cellular uptake and stability while in the circulation (22, 23).

**Figure 1:**
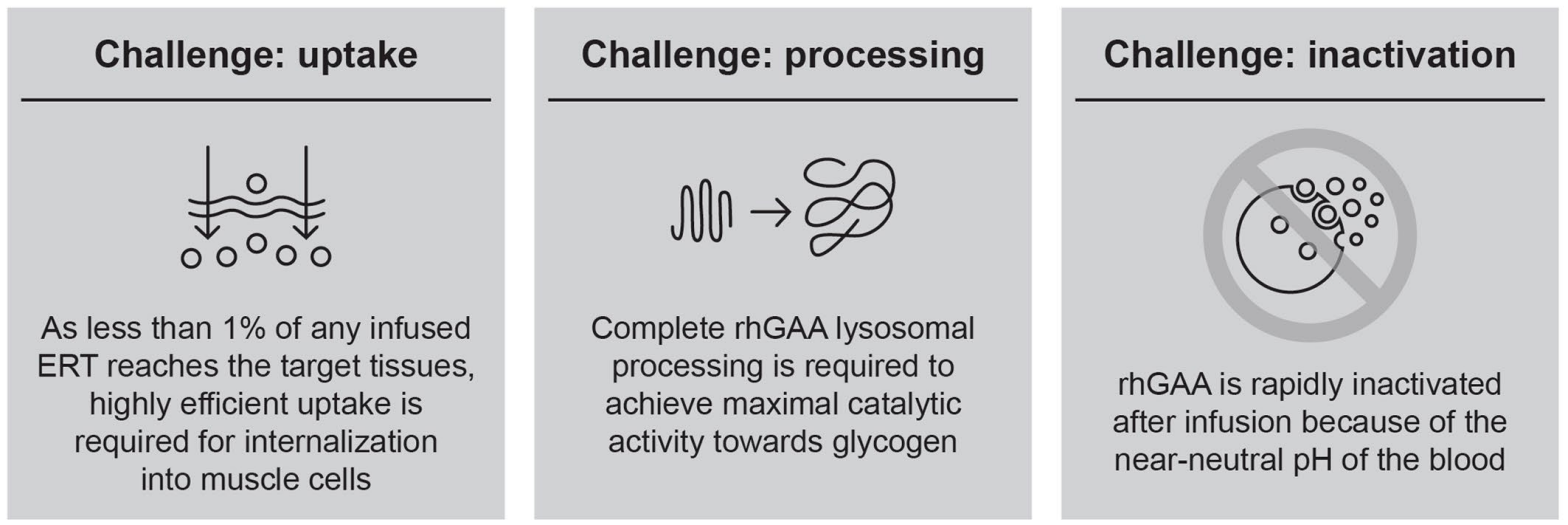
Summary of challenges in delivering rhGAA for Pompe disease.

Over a decade of experience with alglucosidase alfa has demonstrated that the efficacy of this first-generation ERT is constrained by several key challenges (Figure 1), leading to suboptimal clinical outcomes and waning treatment effects over time.

Based on these observations, it has become clear that improvements in ERT-based treatment options must start with developing an rhGAA with specific attributes designed to overcome these key challenges. The first key challenge is efficient uptake into target cells; as less than 1% of an infused rhGAA ERT reaches the interstitial space in muscle because of non-productive clearance by non-target tissues (e.g., liver, spleen), muscle cells are only exposed to ERT concentrations in the low-nanomolar range (24). Thus, highly efficient uptake is required to internalize therapeutic amounts of enzyme into target skeletal and cardiac muscle cells (25). Targeting motor neurons is also desirable; however, the blood–brain barrier prevents penetration of rhGAA ERTs into the central nervous system, and glycogen storage in motor neurons persists even after long-term treatment with rhGAA ERT (26). We therefore focus our report on ERT targeting in skeletal and cardiac muscle.

Cellular uptake of rhGAA is driven by the cation-independent mannose 6-phosphate receptor (CI-MPR; official gene name: *insulin like growth factor 2 receptor* [IGF2R]), a lysosomal delivery receptor that binds bis-M6P, a post-translational modification that is present on a wide assortment of lysosome-fated acid hydrolases. Bis-phosphorylated *N*-glycans, in particular, are responsible for efficient CI-MPR-mediated uptake, with approximately 3,000 times greater affinity for CI-MPR than for monophosphorylated *N*-glycans (25, 27). As alglucosidase alfa contains, on average, only 0.1 mole of bis-phosphorylated *N*-glycans per mole of enzyme, the vast majority of the drug cannot be internalized into target cells at the low resultant enzyme concentrations after dosing (28). Finding effective methods of increasing bis-M6P levels on rhGAA ERT (i.e., without impacting enzyme activity or processing) has been the subject of intense research and presents a viable approach to overcoming this challenge in cellular delivery.

The second key challenge to an effective ERT is complete rhGAA lysosomal processing to allow for maximal catalytic activity. Following cellular uptake through CI-MPR, rhGAA is trafficked through the endocytic pathway and delivered to lysosomes. The single-chain precursor undergoes a series of proteolytic and *N*-glycan-trimming events in the endolysosomal pathway, yielding a mature enzyme with much higher binding affinity for its natural substrate, as evidenced by a 4- to 10-fold reduced Michaelis constant (*K*_m_) for glycogen (29, 30). Importantly, both proteolytic cleavage and *N*-glycan trimming are necessary for full activation after uptake, with either type of processing in isolation providing only a partial (approximately two-fold) reduction in *K*_m_ for glycogen (30). These effects of processing on kinetics are observed only for macromolecular substrates such as glycogen and are not seen with the commonly used fluorogenic small-molecule substrate, 4-methylumbelliferyl *α*-D-glucopyranoside (4-MU-Glc), suggesting that proteolysis and *N*-glycan trimming remove bulky structures proximal to the active site to enable interactions with the large macromolecular substrate (30).

As only small amounts of rhGAA are delivered and internalized into target cells, it is essential that endolysosomal processing proceeds without interruption to yield an enzyme with peak lysosomal activity and therapeutic value. Unfortunately, some synthetic chemistries (e.g., oxime conjugation) that are highly efficient for increasing bis-phosphorylated *N*-glycans on rhGAA are also an obstacle for endogenous glycan-trimming machinery because the linkages that are introduced are very stable and there are no known human enzymes capable of hydrolyzing them (30). These chemistries therefore prevent complete enzyme activation while also negatively impacting unprocessed enzyme activity through steric hindrance (30, 31). Production of rhGAA with naturally increased bis-M6P has thus been proposed as an alternative method for enhancing delivery while preserving the potential for complete post-delivery activation and maximal glycogen hydrolysis activity.

The third challenge in efficiently delivering rhGAA is its inactivation while in the circulation. Even with an engineered enzyme designed to address the uptake and processing challenges, an additional concern is inactivation before delivery to the lysosome. As GAA is an acid hydrolase, it is most stable under acidic conditions and is rapidly inactivated at neutral or alkaline pH (25, 32, 33). This presents a problem for exogenously administered ERTs, which must pass through the near-neutral pH of the blood and interstitium prior to endolysosomal delivery. Denatured (inactivated) enzyme is rapidly eliminated from circulation through non-productive clearance mechanisms (34), contributing to the inefficient cellular delivery of intravenous ERT. Small-molecule stabilizers that transiently bind to ERT but are subsequently displaced in lysosomes present an attractive solution to this problem and could enhance delivery by binding to and stabilizing rhGAA en route to the lysosome.

## 2 Hypothesis

**Figure 2:**
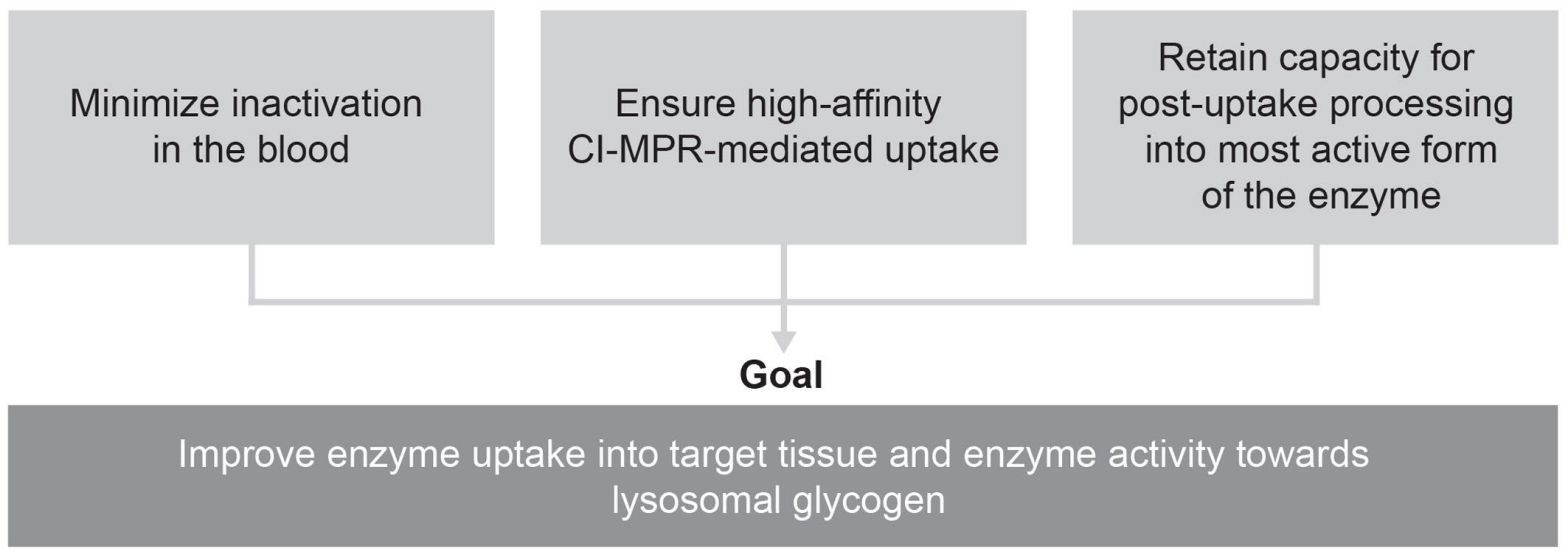
Proposed approach to overcoming key challenges in delivering rhGAA.

Combining an enzyme designed for improved cellular uptake and retained capacity for complete processing with an enzyme stabilizer designed to minimize the inactivation of this enzyme in the bloodstream could help overcome the three key challenges that have thus far limited the efficacy of ERT for Pompe disease (Figure 2).

## 3 Cipaglucosidase alfa: molecular engineering meets ERT

The foundation of any ERT-based treatment for Pompe disease is that the rhGAA protein is able to efficiently enter target cells and the lysosomal compartment. Therefore, efforts to develop next-generation Pompe ERTs have begun with engineering rhGAA for efficient uptake into muscle cells. Cipaglucosidase alfa is a novel, bis-M6P-enriched rhGAA designed for increased cellular uptake through CI-MPR. The enzyme is produced in Chinese hamster ovary (CHO) cells by recombinant DNA technology (25, 35). To generate the stable cell line for rhGAA production, large pools of CHO cells were stably transfected with a DNA vector encoding wild type human GAA. Each transformed CHO cell contained a unique set of untargeted genomic transgene integrations, producing rhGAA with variable co- and post-translational modifications (25). Nearly 1,000 unique rhGAA clones were isolated and screened to identify a clone with an optimal N-glycan profile, including naturally high bis-M6P content for enhanced CI-MPR binding. Since the cell line was generated using a transfection and selection approach based on untargeted (i.e., random) transgene integration, the precise mechanism for the bis-M6P enrichment is unknown.

The selected cellular clone was expanded and a master cell bank was created for use in large-scale production of bis-M6P-enriched rhGAA. A specialized, proprietary, downstream rhGAA-purification process was also developed, which facilitates the highest possible bis-M6P content retention in the finished drug substance.

## 4 Miglustat: a new role as an enzyme stabilizer

Given the rapid inactivation of rhGAA at the near-neutral pH of blood and interstitial space (25, 31–33, 36), there has long been interest in finding a small molecule that can be co-delivered with rhGAA to stabilize the enzyme and enhance target-cell delivery. Miglustat (*N*-butyl-1-deoxynojirimycin) is an iminosugar that mimics the terminal glucose of glycogen, the natural substrate for GAA (25, 37). Miglustat has been used in the clinic for decades in other lysosomal storage disorders (type 1 Gaucher disease and Niemann–Pick disease type C), acting through an alternative mechanism as a substrate reduction therapy at substantially higher doses to inhibit glycosphingolipid synthesis (38–41).

In the context of Pompe disease, miglustat competitively and reversibly binds to the active site of rhGAA and has been shown to effectively stabilize cipaglucosidase alfa while in circulation (25, 36). In a Phase I/II clinical trial (NCT02675465) (42), a 260 mg dose of miglustat was shown to provide a good balance between GAA stabilization and inhibition while in circulation, particularly during the distribution phase of elimination of cipaglucosidase alfa from the systemic circulation. Accordingly, in Phase III studies and product labeling, miglustat is dosed orally (260 mg) once every 2 weeks, 1 h prior to cipaglucosidase alfa infusion. Fasting for 2 h before and 2 h after taking miglustat helps maximize absorption and increase binding to cipaglucosidase alfa while minimizing the potential for adverse events (22, 43).

Importantly, this 260 mg biweekly dose is substantially lower and less frequent than the doses used for treating type 1 Gaucher disease and Niemann–Pick disease type C. When acting as a substrate reduction therapy for either indication, miglustat is taken orally at 100–200 mg three times a day (44, 45). By constraining dosing strictly to 1 h just before each cipaglucosidase alfa infusion, the potential for off-target effects is reduced when using miglustat as an rhGAA stabilizer.

The 260 mg miglustat dose was initially selected based on translational pharmacokinetic (PK)/pharmacodynamic (PD) modeling and simulations from the Pompe (*Gaa* knockout) mouse model. In this analysis, the dose regimen of 260 mg miglustat (human dose equivalent) with 20 mg/kg cipaglucosidase alfa was predicted to provide approximately 18 h of binding and stabilization above the half-maximal inhibitory concentration (IC_50_) in the systemic circulation, with only minimal (approximately 4 h) duration of binding in the lysosome (where the IC_50_ is higher because of the acidic pH). This dose was therefore predicted to provide effective rhGAA stabilization in the blood while minimizing any potential for post-delivery inhibition in the lysosome. Importantly, the intracellular half-life of GAA once delivered to lysosomes is estimated to be nearly 5 days which allows ample time for the dissociation and clearance of miglustat to allow for maximal lysosomal GAA activity. The 260 mg miglustat dose was confirmed as an effective enzyme stabilizer in the first-in-human Phase I/II clinical study (46).

The impact of miglustat on enzyme stabilization is most evident for rhGAAs that are relatively rapidly cleared from circulation, such as cipaglucosidase alfa. The high bis-M6P levels with cipaglucosidase alfa lead to efficient uptake via CI-MPR, while a minor fraction of the enzyme is expected to be cleared by other carbohydrate receptors such as the hepatic asialoglycoprotein receptor, which binds terminal galactose on incompletely processed complex-type *N*-glycans. First-generation rhGAA ERTs contain a higher percentage of complex-type *N*-glycans and much lower bis-M6P content (30), leading to slower clearance kinetics predominantly through non-target receptors. Denaturation of lysosomal enzymes in the neutral pH environment of the blood, resulting in enzyme inactivation and subsequent non-productive clearance, likely impacts the PK of both first- and next-generation rhGAAs. However, the impact of small-molecule stabilization is most apparent for enzymes with faster clearance, whereby there is a proportionally larger exposure impact from rescuing what would otherwise be an inactivated fraction of rhGAA (32).

## 5 Testing the hypothesis: does cipaglucosidase alfa plus miglustat address the key challenges for ERTs in LOPD?

### 5.1 Cipaglucosidase alfa as the foundation of the combination therapy

**Figure 3:**
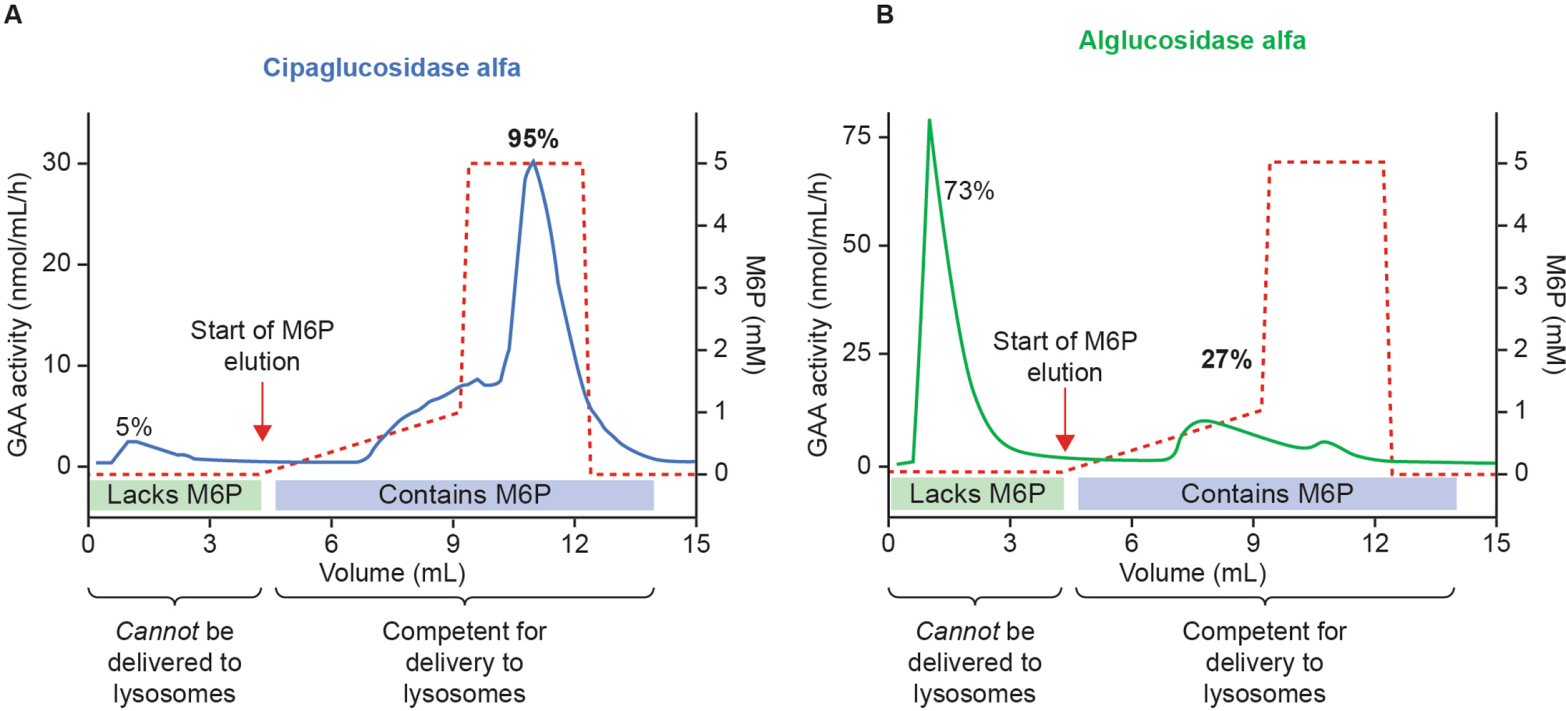
Cipaglucosidase alfa has increased competence for intracellular delivery versus alglucosidase alfa because of enhanced bis-M6P glycosylation. Cipaglucosidase alfa **(A)** or alglucosidase alfa **(B)** was loaded onto a CI-MPR column to assess the relative capacity for each enzyme mixture to interact with the receptor through M6P-bearing *N*-glycans. Following binding, rhGAA was eluted with increasing concentrations of free M6P (dashed red lines). Eluted rhGAA was collected in fractions and levels measured with a 4-MU-*α*-D-glucopyranoside (4-MU-Glc) enzyme activity assay (solid blue and green lines). The vast majority (~95%) of cipaglucosidase alfa bound tightly to the CI-MPR column, eluting only at high M6P concentrations. In contrast, less alglucosidase alfa (27%) bound to the CI-MPR column, reflecting a lack of M6P-bearing *N*-glycans on most enzyme molecules. **(B)** Used with permission from AME Publishing PTE LTD: ‘Challenges in treating Pompe disease: an industry perspective’; Do et al. (24). Permission conveyed through Copyright Clearance Center, Inc.

First, we examined the analytical and PD data – specifically, whether cipaglucosidase alfa results in more bis-M6P than alglucosidase alfa, and whether this leads to enhanced receptor binding and cellular uptake. Mass spectrometry demonstrates that cipaglucosidase alfa contains an average of 1.1–1.3 moles of bis-phosphorylated *N*-glycans per mole of rhGAA (25, 28). In other words, on average, each molecule of enzyme contains at least one copy of the bis-M6P-targeting moiety that is necessary for high-affinity interaction with the CI-MPR uptake receptor. Column chromatography experiments support this assertion, demonstrating that 95% of cipaglucosidase alfa molecules are retained on a CI-MPR column, indicative of their competency in binding the receptor and internalizing into target cells (Figure 3; see Supplementary material for methodology) (25). In contrast, alglucosidase alfa contains only 0.1 moles of bis-phosphorylated *N*-glycans per mole of rhGAA, and the majority (73%) of alglucosidase alfa molecules are not retained on a CI-MPR column, reflecting this lack of M6P and poor potential for target-cell uptake (24, 28).

**Figure 4:**
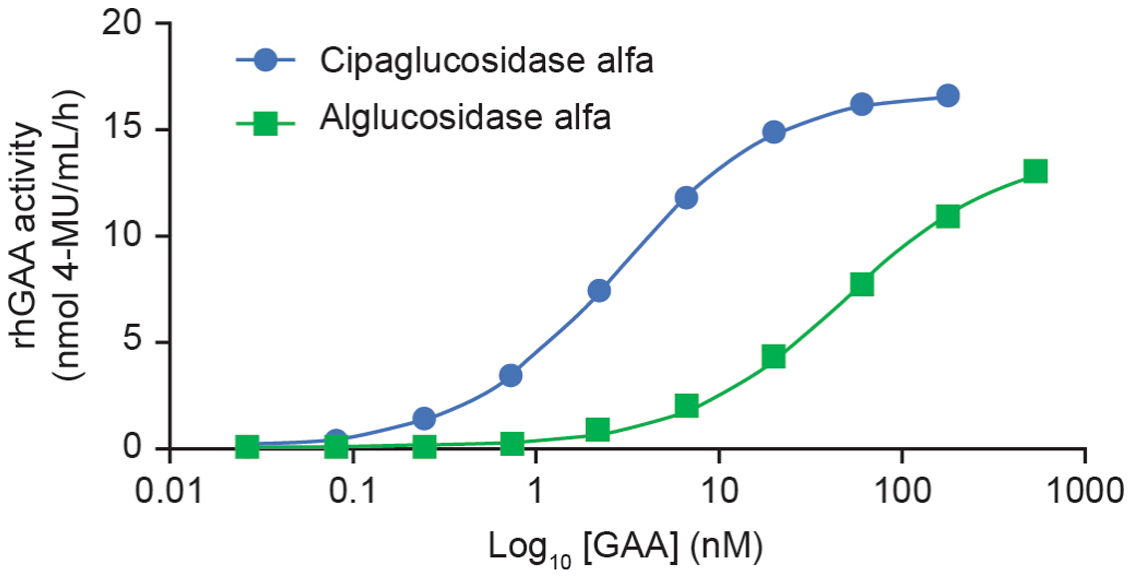
Cipaglucosidase alfa binds to CI-MPR with significantly better affinity than alglucosidase alfa. Cipaglucosidase alfa and alglucosidase alfa were incubated with immobilized soluble CI-MPR receptor in a 96-well plate-based binding assay to determine K_D_. Cipaglucosidase alfa and alglucosidase alfa at concentration ranges of 0.027–180 and 0.74–539 nM, respectively, were incubated with CI-MPR. Unbound GAA was washed from the wells, and binding was quantified by residual GAA enzyme activity *in situ* (see Supplementary material for methodology). The equilibrium dissociation constant, *K*_D_, in nM, was determined from the non-linear regression analysis of the binding curve that yielded the maximum binding (B_max_). *K*_D_ is defined as the concentration of GAA that results in 0.5 B_max_. The *K*_D_ values for cipaglucosidase alfa and alglucosidase alfa are 2.8 and 46.9 nM, respectively. Note that saturation was not achieved for alglucosidase alfa at the concentrations tested, and the true *K*_D_ is most likely to be higher.

This improved capacity for CI-MPR binding was also reflected in a plate binding assay, wherein reversible rhGAA interactions with immobilized CI-MPR were quantified. Here, cipaglucosidase alfa reached peak (saturable) enzyme activity for CI-MPR at concentrations at least 10-fold lower than with alglucosidase alfa (Figure 4), indicative of greatly increased bis-M6P content. The observed binding affinity constants (*K*_D_) for cipaglucosidase alfa and alglucosidase alfa were determined to be ~2.8 and 46.9 nM, respectively, but as saturation was not achieved for alglucosidase alfa at the concentrations tested, the true *K*_D_ is likely substantially higher.

**Figure 5:**
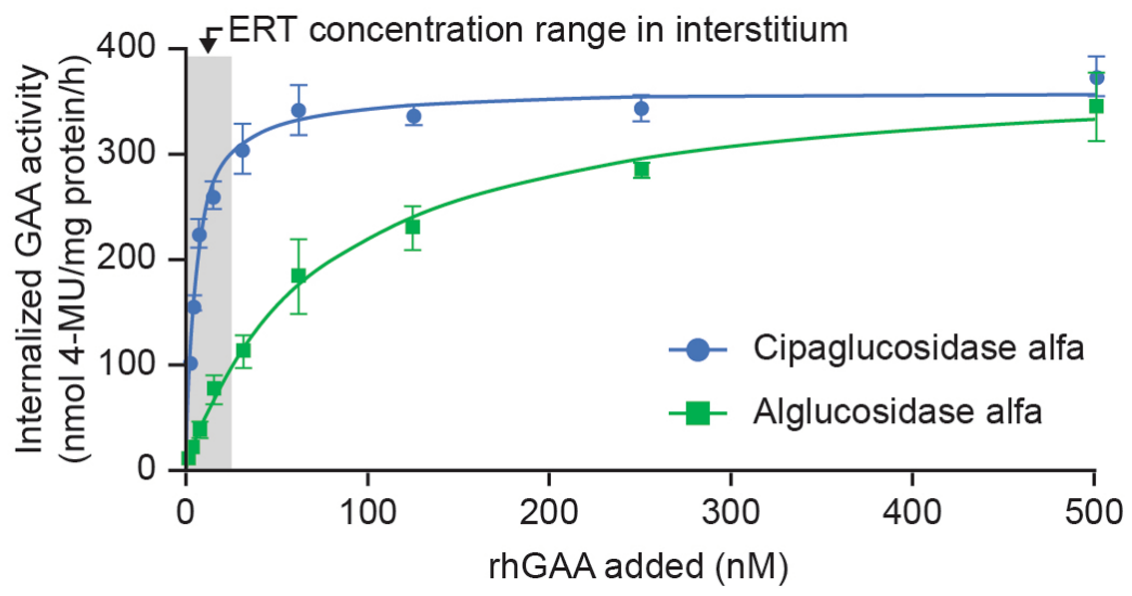
CI-MPR-mediated uptake of cipaglucosidase alfa is more efficient than that of alglucosidase alfa *in vitro.* Cipaglucosidase alfa or alglucosidase alfa at 0–500 nM was incubated with fibroblasts from patients with Pompe disease (see Supplementary material for methodology). Uptake was measured by GAA enzyme activity in cell lysates after washout of unincorporated rhGAA (47). GAA activity was plotted against rhGAA concentration and fitted with a one-site binding with saturation model from which maximal uptake and K_uptake_ (the concentration of rhGAA that yields half-maximal uptake) were calculated by non-linear regression analysis. The shaded area represents the estimated approximate concentration of rhGAA ERT in the interstitial space available for uptake into cells (24).

As expected, this improved binding to CI-MPR leads to enhanced cellular delivery. In cell-uptake experiments using fibroblasts from individuals with Pompe disease, cipaglucosidase alfa reached peak (saturable) enzyme activity at lower concentrations than alglucosidase alfa, indicative of more efficient uptake because of the higher affinity of cipaglucosidase alfa for CI-MPR (Figure 5). Notably, large differences in uptake were observed in the low nanomolar ranges that are expected in the interstitium after intravenous dosing, with cipaglucosidase alfa achieving near-maximal levels of uptake, compared with only minimal uptake for alglucosidase alfa. In these experiments, the half-maximal extracellular concentration for cellular uptake for cipaglucosidase alfa was 5–15 nM, as opposed to >125 nM for alglucosidase alfa.

### 5.2 Clinical studies further support improved uptake for cipaglucosidase alfa in individuals living with Pompe disease

**Figure 6:**
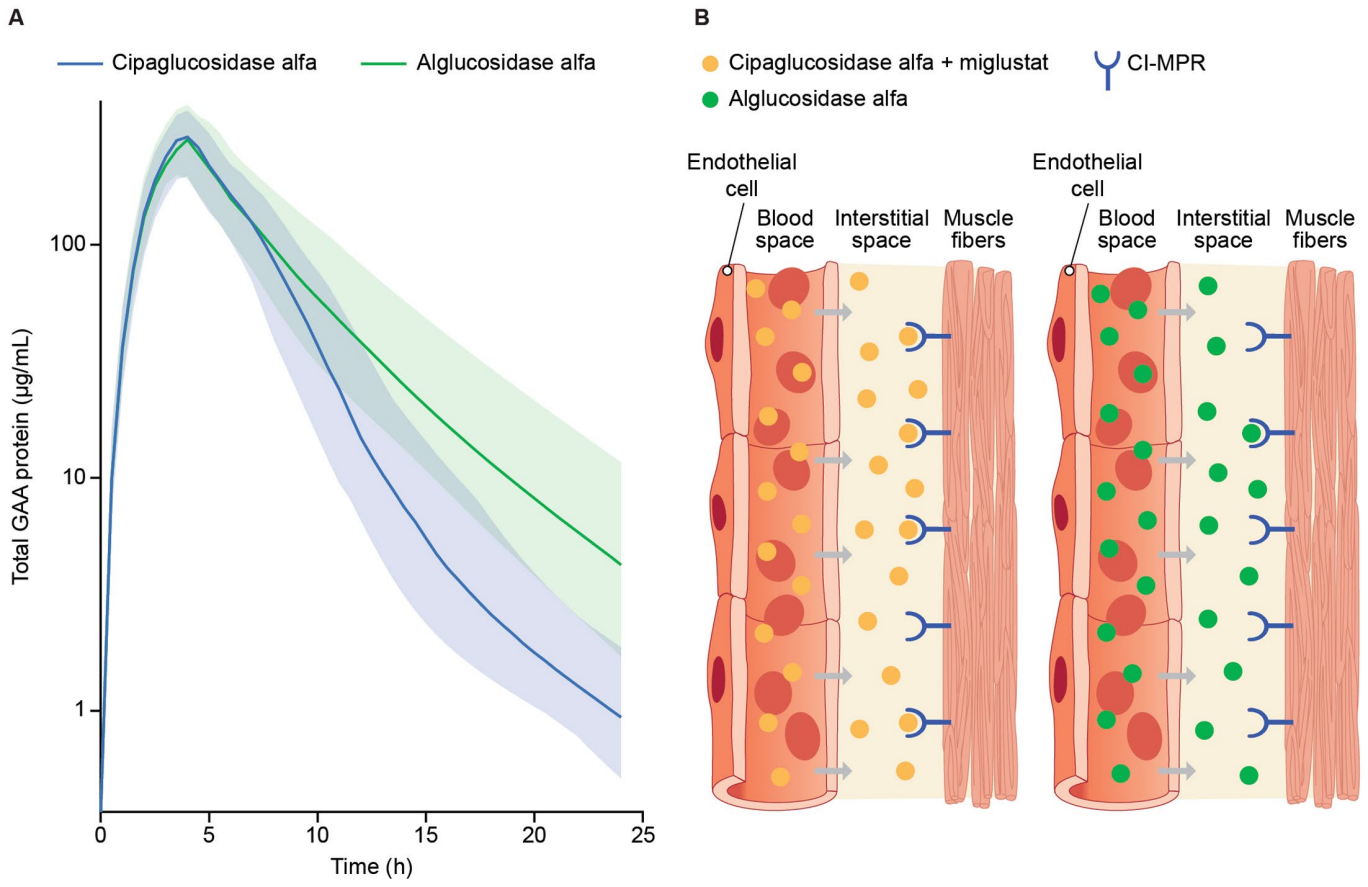
**(A)** Model-predicted plasma GAA protein profiles for participants in the PROPEL trial and **(B)** infographic representation of rhGAA uptake from the bloodstream into muscle. **(A)** Total GAA protein was measured by signature peptide mass spectrometry at week 52, just before the start of GAA infusion (time 0) and at 1, 4, 6, 12, and 24 h after infusion. A population PK model is shown, with solid lines reflecting predicted median total GAA protein concentrations and shaded regions indicating 5th and 95th percentiles (47). While both drugs reached peak concentrations by approximately the end of infusion (4 h), predicted median total GAA concentrations declined at a higher rate for cipaglucosidase alfa during the distribution phase of terminal elimination, consistent with greater bis-M6P levels facilitating more efficient uptake through the CI-MPR receptor. **(B)** rhGAA distribution from systemic circulation to the interstitial space in muscle tissue, with cipaglucosidase alfa plus miglustat shown on the left and alglucosidase alfa shown on the right. While similar concentrations of both drugs are achieved shortly after administration in blood, the increased bis-M6P content of cipaglucosidase alfa facilitates higher-affinity interaction with the target CI-MPR receptor, leading to faster clearance and greater uptake into muscle fibers. Used with permission from AME Publishing PTE LTD: ‘Challenges in treating Pompe disease: an industry perspective’; Do et al. (24). Permission conveyed through Copyright Clearance Center, Inc.

Data collected as part of the PROPEL trial (NCT03729362) showed that both cipaglucosidase alfa and alglucosidase alfa reached peak concentrations in the blood near the end of infusion (4 h), which steadily declined over 24 h after infusion. Clearance rates, however, were notably different, with cipaglucosidase alfa exhibiting faster clearance from blood plasma (Figure 6A), with a consequent smaller area under the concentration–time curve (AUC).

The generally greater alglucosidase alfa mean AUC is driven by higher terminal phase concentrations due to slower plasma clearance. Cipaglucosidase alfa was shown to have a non-linear clearance pathway in addition to the linear clearance observed for both enzymes. Although a non-linear clearance pathway was tested in the alglucosidase alfa population PK model, this added clearance pathway did not improve model fit and was thus omitted from the final model (47). The increased rate of cipaglucosidase alfa clearance from circulation and improved glycosylation with enhanced bis-M6P levels for CI-MPR uptake into muscle suggest improved target-mediated drug disposition (Figure 6B).

### 5.3 Cipaglucosidase alfa retains the capacity to undergo complete processing and activation following cellular uptake

**Figure 7:**
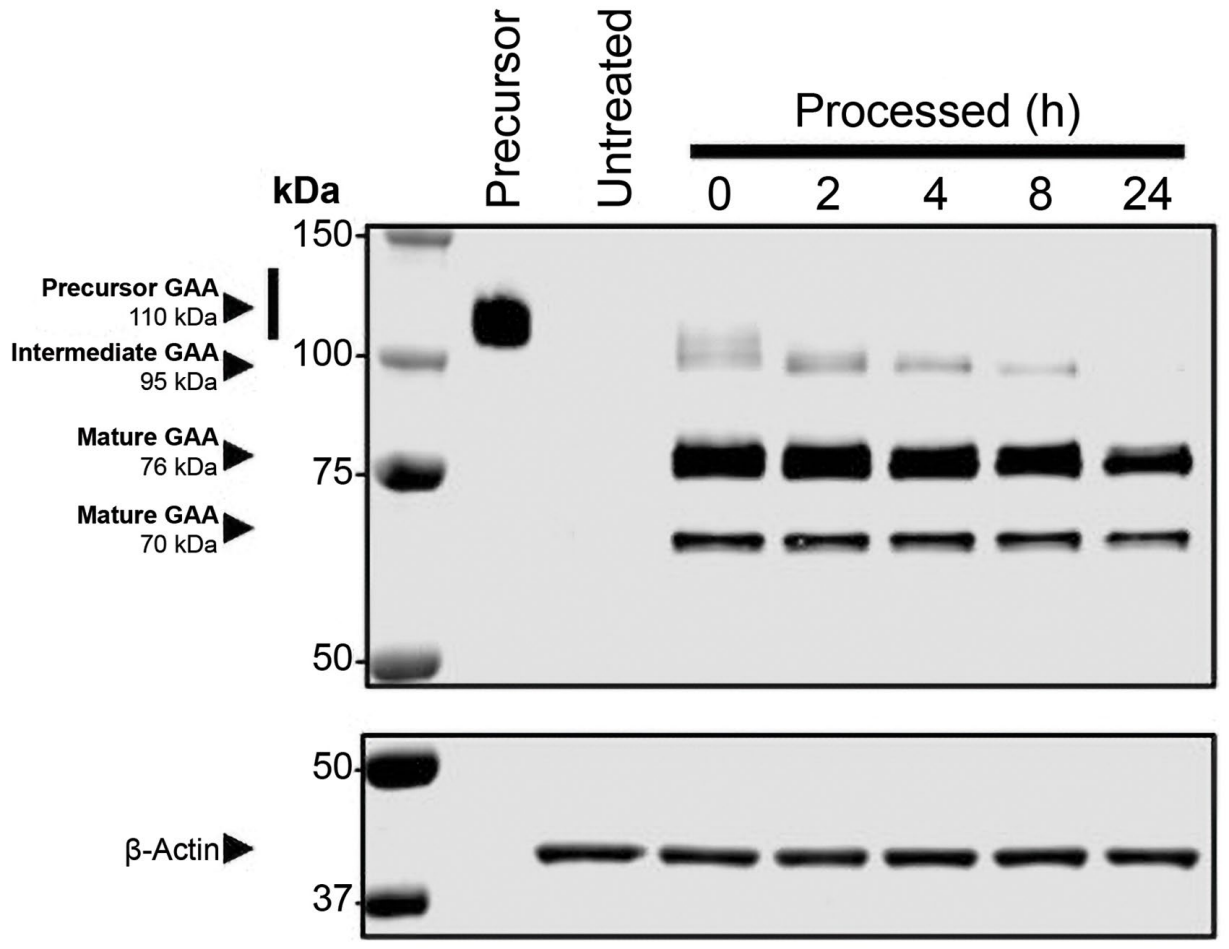
Cipaglucosidase alfa undergoes full proteolytic processing *in cellulo.* Fibroblasts from individuals with Pompe disease were treated with 0 (untreated) or 20 nM cipaglucosidase alfa for 18 h at 37°C. After 18 h, the uptake medium was replaced with growth medium, and cells were harvested at the indicated time points (see Supplementary material for methodology). The Western blot shows analysis of rhGAA, processed over a 24 h time course, compared with precursor enzyme (lane 1). The blot was probed with a primary antibody against GAA (top) or actin (bottom). Precursor, intermediate, and mature bands are highlighted by arrows, and time points of harvest are shown above each lane.

Studies have shown that rhGAA undergoes proteolytic and *N*-glycan-trimming events in the endolysosomal pathway that result in a 4- to 10-fold improvement in affinity for glycogen, depending on the assay conditions used (29, 30). Importantly, complete maturation of cipaglucosidase alfa was observed over 24 h after uptake in Pompe patient fibroblasts, confirming that the enzyme is fully activated following delivery (Figure 7). Furthermore, as cipaglucosidase alfa contains only endogenous *N*-glycan structures with natural bis-M6P, glycan trimming is not impeded, as was observed for some synthetic strategies for bis-M6P conjugation (e.g., chemical conjugation of bis-M6P via oxime bonds to *N*-glycan terminal sialic acids, as used for avalglucosidase alfa) (30).

### 5.4 Miglustat reduces inactivation to maximize the availability of circulating active enzyme for target-cell uptake

While the glycoengineering used to create cipaglucosidase alfa solves the challenges of uptake and processing, the enzyme is still an acid hydrolase and vulnerable to inactivation in the near-neutral pH of blood. Co-administration with miglustat aims to address this challenge by binding to cipaglucosidase alfa while in circulation. Here, we examine preclinical and clinical data supporting this stabilizing activity.

In published *in vitro* and *in cellulo* studies, co-incubation of fibroblasts from individuals with Pompe disease with recombinant rhGAA and a small-molecule stabilizer improved the stability of the enzyme, ultimately increasing the amount of enzyme that was delivered to lysosomes and processed into the mature form (48). Similar stabilizing activity has also been documented for miglustat in human blood (*ex vivo*) at concentrations comparable with those achieved in the bloodstream following clinical dosing (25, 47).

Importantly, this activity translates into measurable PK improvements for rhGAA ERT *in vivo*. In preclinical models, miglustat stabilized rhGAAs, including cipaglucosidase alfa, increasing exposure and prolonging the biodistribution half-life in blood (25, 36). Toxicokinetic studies in non-human primates showed that oral administration of miglustat increased the exposure of cipaglucosidase alfa approximately two-fold (25). Smaller but still statistically significant effects were also observed in rodents, in which miglustat increased cipaglucosidase alfa exposure by up to 25%, particularly during the distribution terminal elimination phase (25).

**Figure 8:**
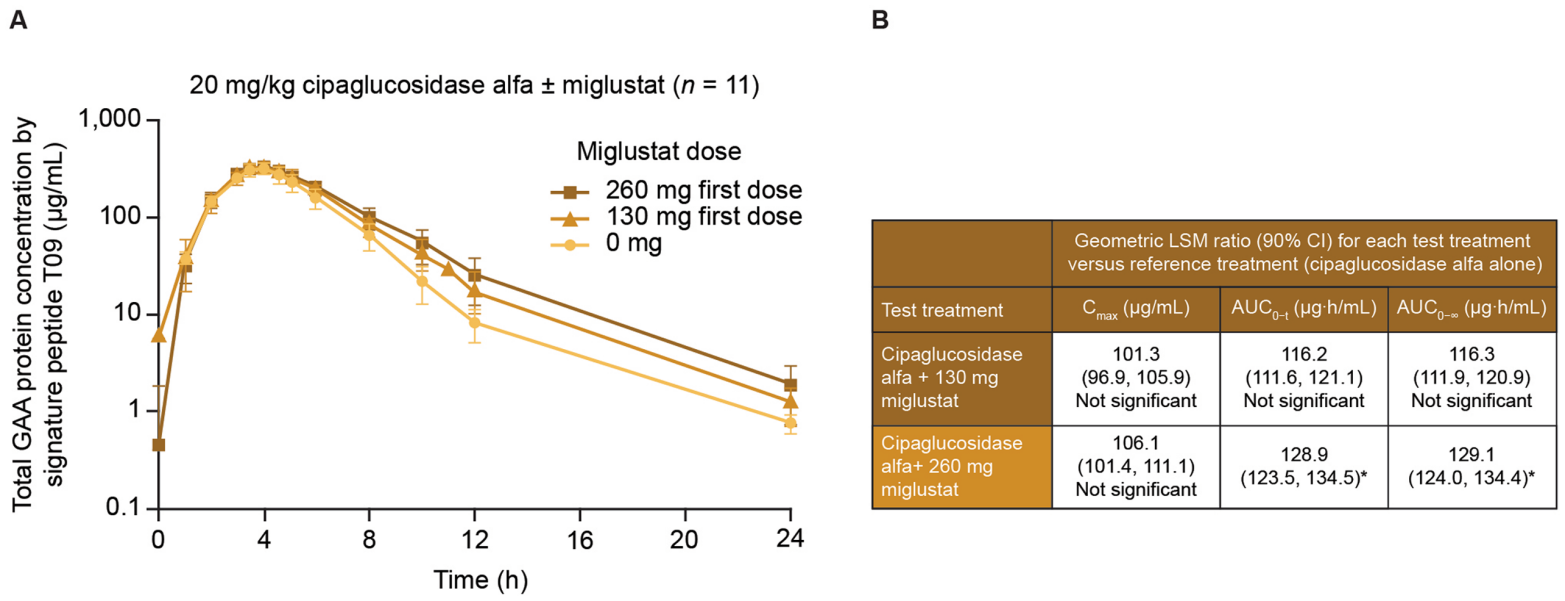
Miglustat increases cipaglucosidase alfa exposure in adults with Pompe disease. Individuals with LOPD from study ATB200-02 (NCT02675465; *n* = 19) were treated with a single dose of 20 mg/kg intravenous cipaglucosidase alfa alone or co-administered with 130 mg or 260 mg miglustat. Blood samples were collected just before the start of infusion and at 1, 2, 3, 3.5, 4, 4.5, 5, 6, 8, 10, 12, and 24 h after infusion. **(A)** Total rhGAA protein levels were measured in plasma with a validated liquid chromatography–tandem mass spectrometry signature peptide assay. While similar peak rhGAA concentrations were observed with and without miglustat, miglustat increased cipaglucosidase alfa exposure in the distribution phase in a dose-dependent manner. **(B)** Data were analyzed with ANOVA and geometric LS mean ratios for C_max_, AUC_0–t_, and AUC_0–∞_. Neither parameter was significantly increased for cipaglucosidase alfa with 130 mg miglustat versus cipaglucosidase alfa alone. However, the geometric LS mean ratios for AUC_0–t_ and AUC_0–∞_ indicated that cipaglucosidase alfa with 260 mg miglustat was significantly increased from that for cipaglucosidase alfa alone, while C_max_ was not. **(A)** Adapted from Byrne et al. (46), used under CC BY 4.0 (https://creativecommons.org/licenses/by/4.0/). *An upper limit of 90% CI >125% indicated a statistically significant increase from the reference treatment. ANOVA, analysis of variance; AUC_0–t_, AUC from time zero to the last quantifiable time point; AUC_0–∞_, AUC from time zero to infinity; CI, confidence interval; C_max_, maximum concentration; LS, least-squares.

In a Phase I/II trial of cipaglucosidase alfa plus miglustat in ERT-experienced individuals living with LOPD (49), miglustat increased the distribution half-life (*α* phase) of 20 mg/kg cipaglucosidase alfa in a dose-dependent manner, with a 260 mg dose increasing the half-life by 46.7%, consistent with a stabilizing effect in plasma. This was accompanied by statistically significant increases in the exposure partial AUC of 43.6% for the 260 mg dose from the period spanning peak plasma levels to 24 h post-dose (AUC_tmax–24 h_; Figure 8).

From time zero to the last quantifiable time point, miglustat increased plasma GAA exposure 28.5% (AUC_0–t_), which is higher than the approximately 7% increase observed in Pompe (*Gaa* knockout) mice at a similar dose (10 mg/kg miglustat, Table 1), supporting the translatability of rhGAA plus miglustat findings in mice.

**Table 1:** Miglustat increases rhGAA exposure in humans to a slightly greater extent than that observed in mice.

Species	Cipaglucosidase alfa dose	Miglustat dose	Cipaglucosidase alfa AUC_0–t_	Cipaglucosidase alfa AUC_0–t_ with miglustat	Increase, %
Gaa −/− mice	20 mg/kg	10 mg/kg	14.6 (×10^4^ h [nmol/mL/h])	15.6 (×10^4^ h [nmol/mL/h])	6.8
Human	20 mg/kg	260 mg (~10 mg/kg)	1,410 μg × h/mL	1812 μg × h/mL	28.5

Gaa −/− mice (*n* = 5/group) were administered cipaglucosidase alfa with or without miglustat and plasma rhGAA AUC_0–t_ was measured with an enzyme activity assay and reported as units of hydrolyzed 4-MU-Glc. Individuals with Pompe disease (ERT-switch ambulatory patients, *n* = 11) were administered cipaglucosidase alfa with or without miglustat and ERT exposure (AUC) was measured with an LC–MS/MS rhGAA signature peptide assay and reported as mass of protein. Miglustat increased AUC_0–t_ by 6.8% in mice and 28.5% in humans. Table adapted with permission from The American Society for Pharmacology and Experimental Therapeutics, from Brudvig et al. (51), permission conveyed through Copyright Clearance Center, Inc. LC–MS/MS, liquid chromatography–tandem mass spectrometry.

**Figure 9:**
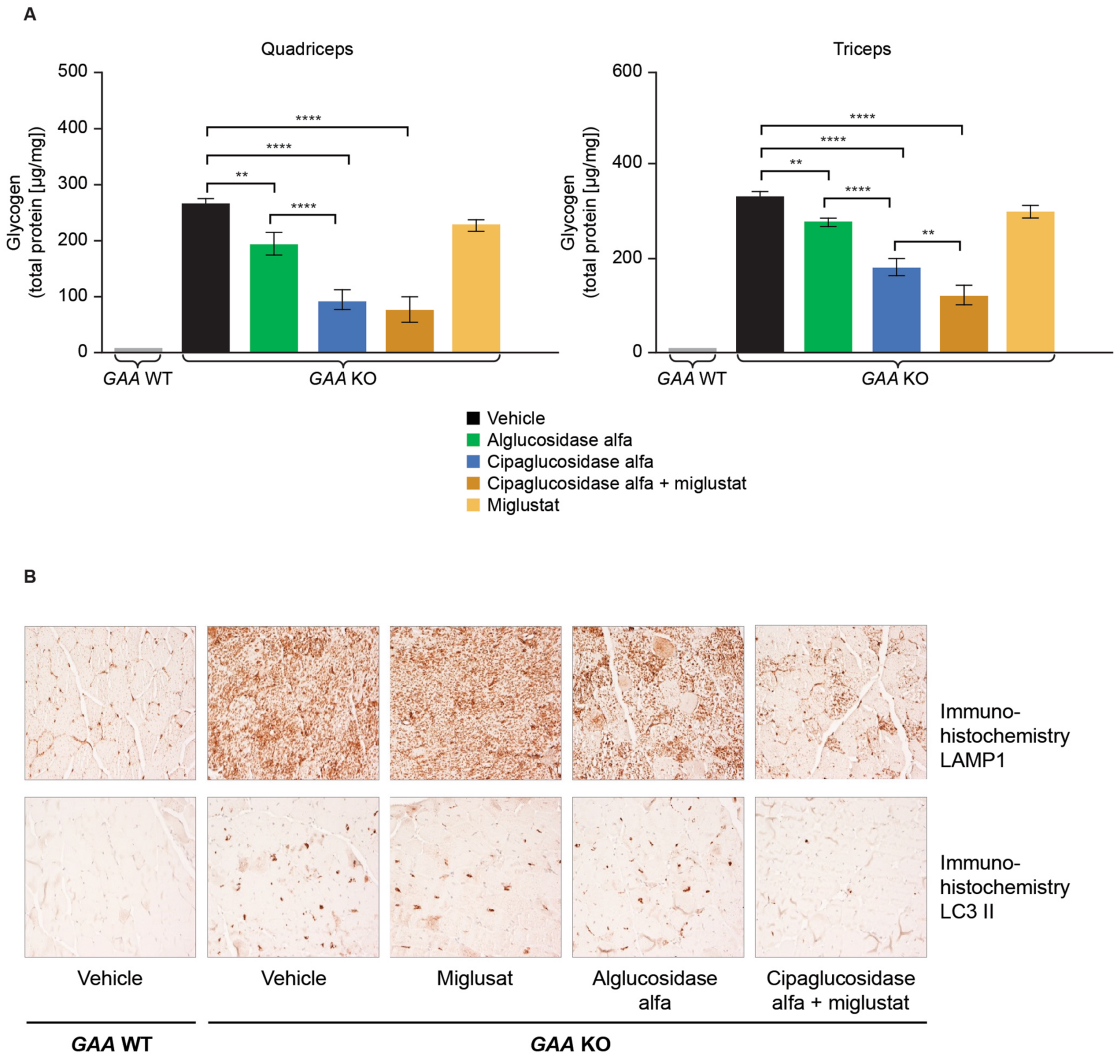
**(A)** Co-administration of miglustat with cipaglucosidase alfa improved treatment outcomes compared with vehicle control, whereas miglustat alone had no impact on glycogen reduction in the muscles of *Gaa* −/− mice, and **(B)** no reduction in LAMP1 or LC3 II was observed in *Gaa* −/− mice treated with miglustat alone. **(A)** *Gaa* −/− mice (15–19 weeks old; *n* = 8/group) received two biweekly IV administrations of vehicle, 20 mg/kg alglucosidase alfa, 20 mg/kg cipaglucosidase alfa + 10 mg/kg miglustat (administered orally 30 min prior to cipaglucosidase alfa IV injection), or 10 mg/kg miglustat alone (30). Tissues were collected 14 days after the second administration, homogenized, and assayed for glycogen content. Glycogen was significantly reduced in the quadriceps and triceps of animals treated with cipaglucosidase alfa + miglustat compared with vehicle-treated animals and those treated with miglustat alone. One-way ANOVA with Fisher’s least significant difference *post hoc* test: *p < 0.05; **p < 0.005; ****p < 0.00005. **(B)** *Gaa* −/− mice (15–19 weeks old; *n* = 8/group) received two biweekly IV administrations of vehicle, 20 mg/kg alglucosidase alfa, 20 mg/kg cipaglucosidase alfa + 10 mg/kg miglustat (administered orally 30 min prior to cipaglucosidase alfa IV injection), or 10 mg/kg miglustat alone (30). Tissues were collected 14 days after the second administration and qualitatively analyzed with immunohistochemistry. Accumulation of LAMP1-positive lysosomes is a pathological hallmark of LOPD. LC3 II localizes to autophagosome membranes, and LC3 II positive aggregates in vehicle-treated *Gaa* −/− mice reflect autophagic build-up. Cipaglucosidase alfa plus miglustat-treated mice displayed a reduction in LAMP1 and LC3 II signals in quadriceps compared with alglucosidase alfa treatment, and no reduction was observed in mice treated with miglustat alone. Representative images from seven or eight animals analyzed per treatment group, 200x magnification. IV, intravenous; KO, knockout; LAMP1, lysosomal-associated membrane protein 1; LC3, microtubule-associated protein light chain 3; WT, wild type.

In the context of therapy for Pompe disease, the sole function of miglustat is to stabilize cipaglucosidase alfa while in circulation. When dosed orally prior to rhGAA infusion, the concentration of miglustat in the plasma increases dose proportionally after administration, and miglustat is then rapidly eliminated and excreted. Miglustat competitively binds the active site of the enzyme and transiently inhibits activity while it is being distributed to the target tissues. Still, it is not expected to meaningfully inhibit enzyme activity in the acidic environment of the lysosome, where inhibitory potency is much lower. In humans, miglustat has a relatively short terminal elimination half-life in plasma of 6 h concurrent with rapid renal excretion (50). Therefore, binding with cipaglucosidase alfa is maintained in plasma for approximately 24 h post start of infusion, during which time both miglustat and cipaglucosidase alfa decline in tandem to negligible concentrations. However, due to its rapid elimination from both plasma and tissues, along with decreased inhibitory potential in the lysosome, miglustat declines precipitously after only approximately 4 h in target tissues post start of infusion. Importantly, rhGAA has a much longer intracellular half-life of nearly 1 week after cellular uptake such that the ERT has ample time for glycogen hydrolysis. Preclinical studies support a lack of relevant activity for miglustat alone; repeated dosing had no significant impact on pathological measures in *Gaa* −/− mice, including muscle glycogen levels and immunohistochemical markers of lysosomal mass and autophagic build-up (Figure 9). A recent preclinical study suggested that miglustat did not affect the outcomes in GAA KO mice when administered with alglucosidase alfa and avalglucosidase alfa (32). These results are in conflict with published data for alglucosidase alfa and cipaglucosidase alfa, and the potential reasons for these differences have been discussed in detail (32, 51, 52). Unlike prior studies which have consistently found PK and PD benefits for miglustat when administered alongside rhGAAs (25, 36, 47), this study utilized a weekly dosing scheme that likely obscured the benefits of stabilization. For alglucosidase alfa, stabilization appeared to be of minimal impact in the context of poor target tissue enzyme uptake owing to low bis-M6P levels. For avalglucosidase alfa, weekly dosing may have saturated target tissues with enzyme, leaving little room for improvement through stabilization during delivery.

### 5.5 Treatment with cipaglucosidase alfa plus miglustat yields improved pathological and functional outcomes

**Figure 10:**
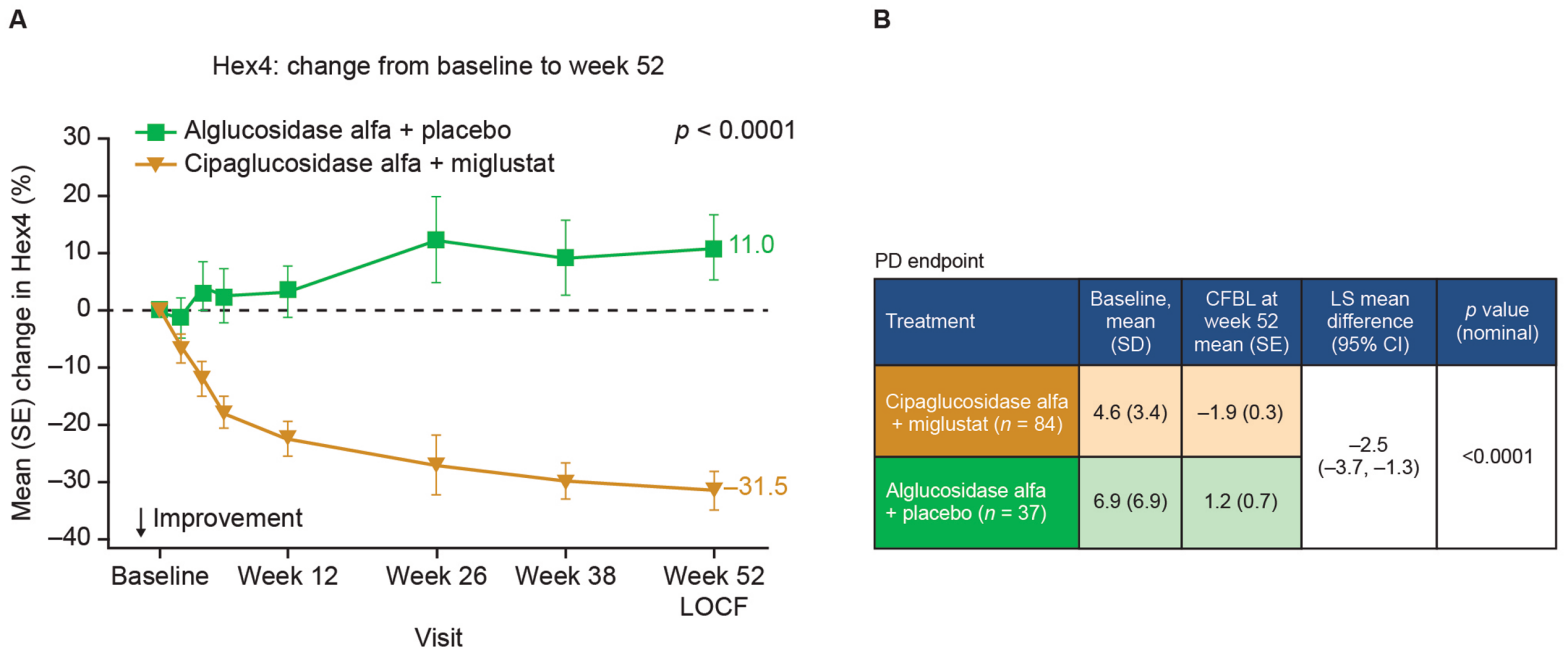
Hex4 levels are improved in patients treated with cipaglucosidase alfa plus miglustat compared with alglucosidase alfa plus placebo. Hex4 is a breakdown product of glycogen and biomarker surrogate for glycogen storage metabolism. **(A)** Percentage CFBL in urine Hex4 in the combined PROPEL study population (ERT-experienced plus ERT-naïve patients). Week 52 data LOCF (55). **(B)** Hex4 (mmol/mol creatinine) CFBL calculations at week 52 performed using an ANCOVA model with LOCF, with associated LS mean difference and nominal *p* value. Figure adapted from Schoser et al. (55), with permission from Elsevier. ANCOVA, analysis of covariance; CFBL, change from baseline; LOCF, last observation carried forward; SD, standard deviation; SE, standard error.

Given the PK benefits observed for cipaglucosidase alfa versus alglucosidase alfa and the additional benefits obtained by stabilization with miglustat, the combination of cipaglucosidase alfa plus miglustat should be expected to improve pathological outcomes. In *Gaa* −/− mice, the increased uptake of cipaglucosidase alfa administered alone and, to a slightly greater extent, with miglustat translates into improved muscle glycogen clearance compared with alglucosidase alfa (Figure 9A) (25). Similarly, adults with LOPD in the PROPEL trial exhibited greater reductions in circulating hexose tetrasaccharide (Hex4), a breakdown product of glycogen, when treated with cipaglucosidase alfa plus miglustat versus alglucosidase alfa (Figure 10).

**Figure 11:**
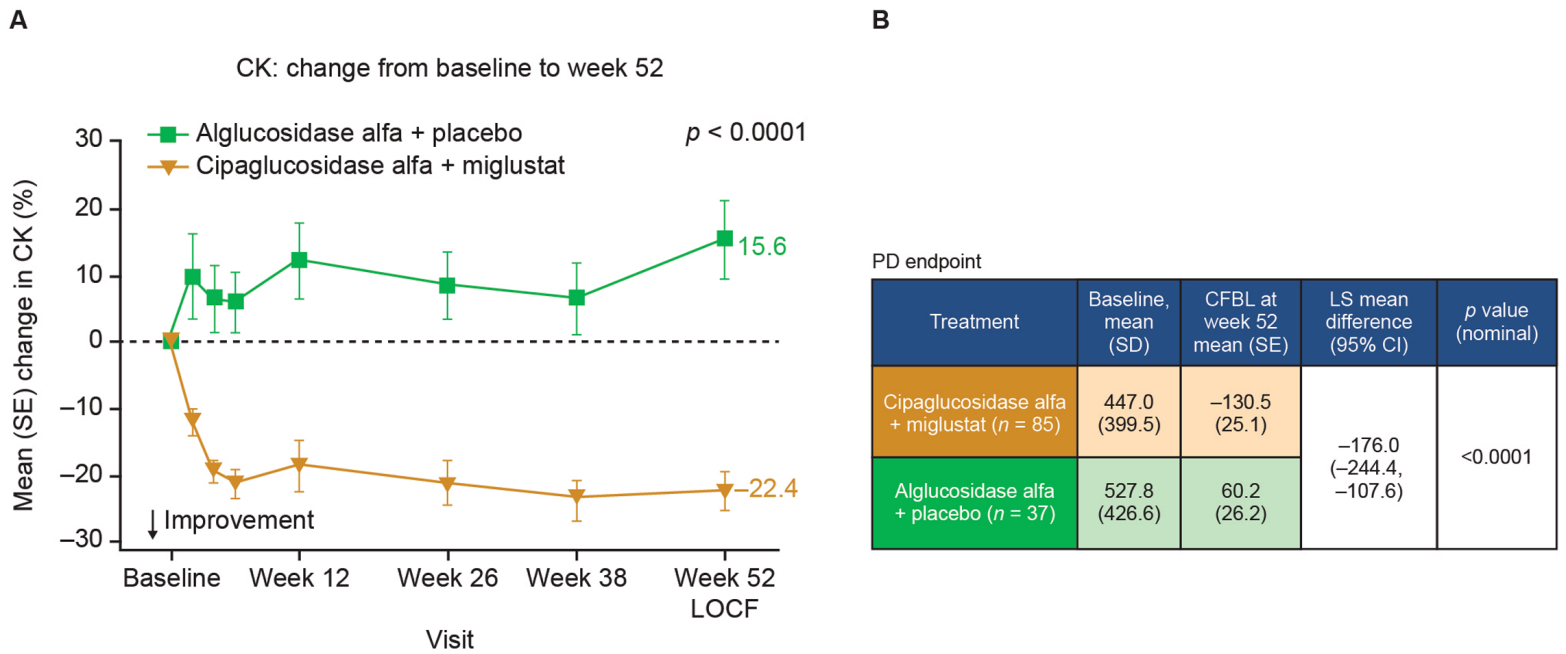
CK levels are reduced in patients with Pome disease treated with cipaglucosidase alfa plus miglustat compared with those treated with alglucosidase alfa plus placebo. **(A)** Percentage CFBL in serum CK in the combined PROPEL study population (ERT-experienced plus ERT-naïve patients). Week 52 data LOCF. **(B)** CK (U/L) CFBL calculations at week 52 performed using an ANCOVA model with LOCF, with associated LS mean difference and nominal *p*-value. Figure adapted from Schoser et al. (55), with permission from Elsevier.

In Pompe disease, lysosomal glycogen accumulation eventually leads to muscle cell damage and degeneration. Creatine kinase (CK), a muscle-enriched enzyme, is released into the blood when the muscle is degenerating and is used as a diagnostic and monitoring biomarker for LOPD (53, 54). In the PROPEL trial, adults with LOPD receiving cipaglucosidase alfa plus miglustat exhibited greater reductions in CK from baseline to week 52 than did patients receiving alglucosidase alfa (Figure 11), suggesting attenuation of muscle degeneration.

**Figure 12:**
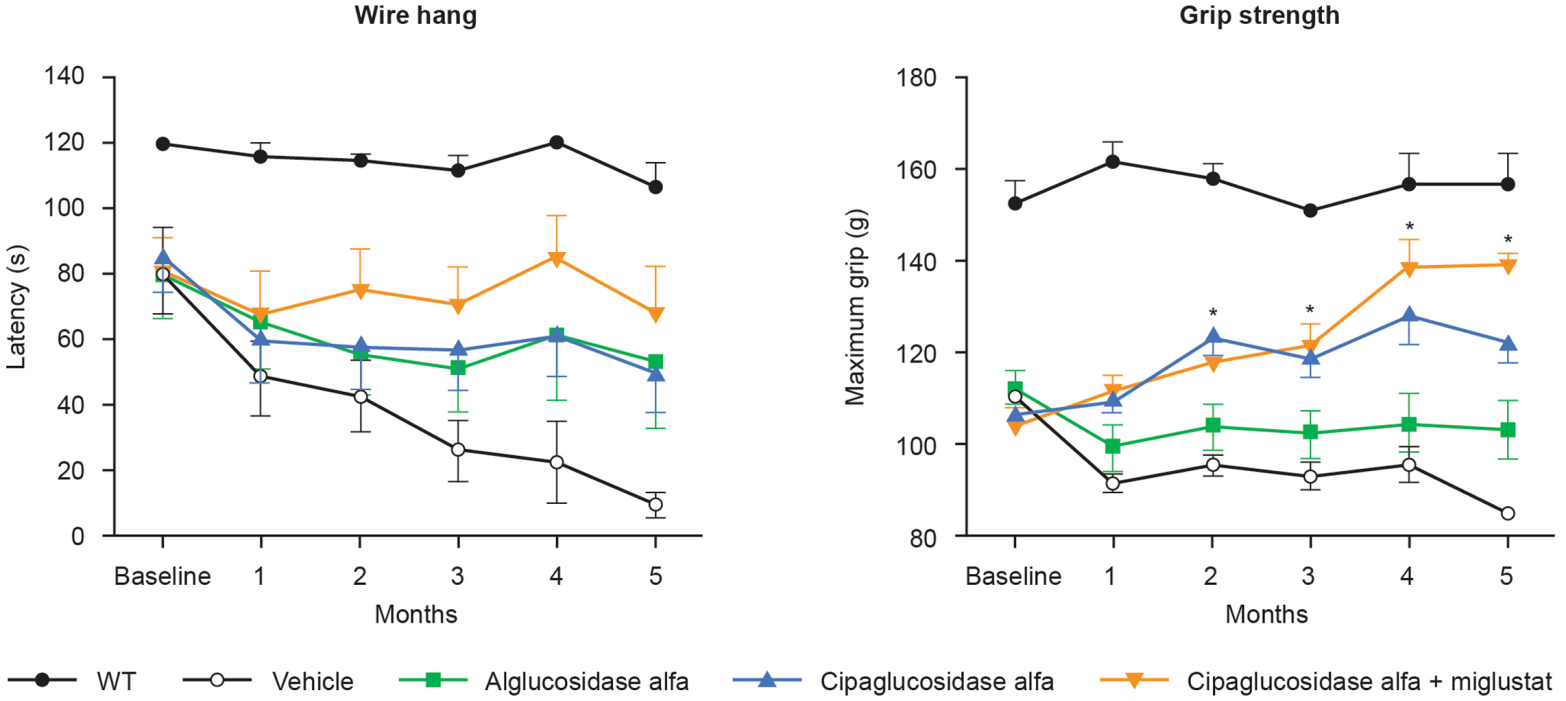
Muscle-function assessments (wire hang and grip strength) in *Gaa* −/− mice. *Gaa* −/− mice (15–19 weeks old; *n* = 8/group) received biweekly IV administrations of vehicle, 20 mg/kg alglucosidase alfa, 20 mg/kg cipaglucosidase alfa alone, or 20 mg/kg cipaglucosidase alfa + 10 mg/kg miglustat (administered orally 30 min prior to cipaglucosidase alfa IV injection). Muscle function was assessed once a month for 5 months, 7 days after administration of drug. The wire-hang latency assay (left) was conducted once on two consecutive days; the average of two assessments is shown. Maximum grip strength (right) was measured three times on the same day; the average of those three assessments is shown. Each point represents mean ± SE of 15 animals/group up to 3 months and eight animals/group for the remaining 3 months (seven animals sacrificed after 3 months). One-way ANOVA with Dunnett’s *post hoc* test. *p < 0.05 versus alglucosidase alfa. Figure adapted from Xu et al. (25), used under CC BY 4.0 (https://creativecommons.org/licenses/by/4.0/).

Most importantly, treatment with cipaglucosidase alfa plus miglustat has demonstrated improved functional outcomes. In preclinical studies, *Gaa* −/− mice treated with cipaglucosidase alfa plus miglustat exhibited improved muscle function in the wire-hang and grip-strength assays that further enhance the already large improvements achieved by cipaglucosidase alfa alone (Figure 12). In these studies, miglustat increased ERT exposure (AUC_0–t_) by 6.8%, which is slightly lower than the 9% increase observed in humans, suggesting that the addition of miglustat to cipaglucosidase alfa should offer a similar incremental functional benefit when used as a treatment for Pompe disease in humans.

**Figure 13:**
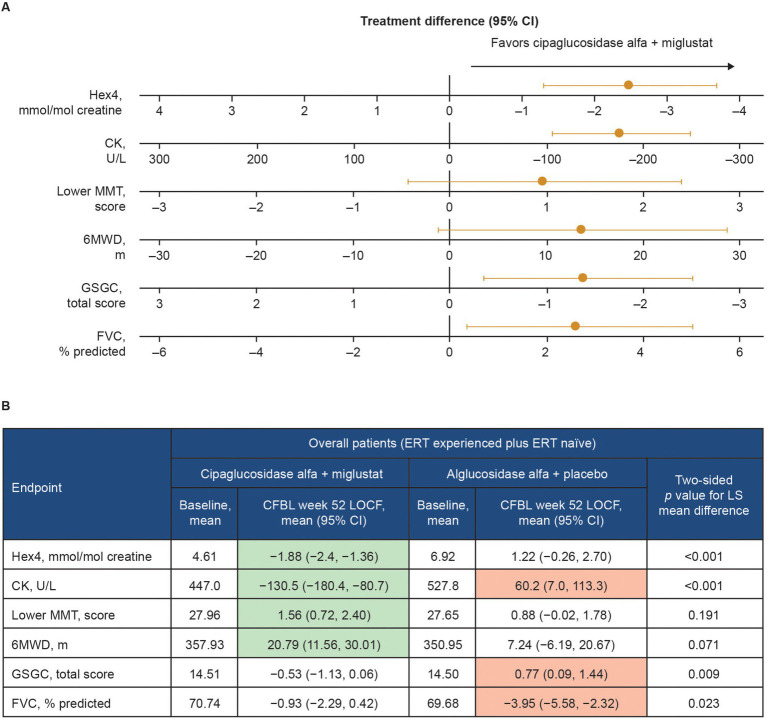
Change from baseline at week 52 of PROPEL – effect of cipaglucosidase alfa plus miglustat compared with alglucosidase alfa plus placebo in key efficacy outcomes. **(A)** Forest plot illustrating mean estimated treatment differences between cipaglucosidase alfa plus miglustat versus alglucosidase alfa plus placebo and corresponding 95% CIs are shown for the combined PROPEL study population for each outcome, with units as indicated on the x-axes. For all outcomes, right-sided directionality of treatment differences indicates favorable outcomes for cipaglucosidase alfa plus miglustat compared with alglucosidase alfa plus placebo. **(B)** The table shows baseline mean values and Week 52 CFBL values for cipaglucosidase alfa plus miglustat and alglucosidase alfa plus placebo. Shaded CFBL indicates nominally significant improvement (green) or nominally significant worsening (red) from baseline (i.e., the 95% CI does not include zero) within each treatment group. The *p*-values (two-tailed LS mean difference) shown in the far-right column are for the between-group treatment differences illustrated in the forest plot.

Functional benefits of combination therapy have also been observed in multiple clinical studies. In a Phase I/II clinical trial (NCT02675465), patients with LOPD treated with cipaglucosidase alfa plus miglustat had durable improvements in motor function and biomarker levels from baseline up to 48 months of follow-up (46). In the subsequent Phase III PROPEL trial (NCT03729362), analyses of the least squares (LS) mean difference in change from baseline to week 52 for the primary and key secondary endpoints indicated a favorable effect of treatment with cipaglucosidase alfa plus miglustat over alglucosidase alfa on multiple measures in the overall population of ERT-experienced plus ERT-naïve patients (Figure 13). In this combined population, a nominally significant improvement from baseline was observed for the cipaglucosidase alfa plus miglustat group for Hex4 and CK levels, manual muscle test (MMT), and 6-min walk distance (6MWD), while a nominally significant worsening from baseline was observed for the alglucosidase alfa plus placebo group for CK levels, Gower’s Maneuver, Stairs, Gait, Chair (GSCG), and forced vital capacity (FVC; Figure 13) (55). Additional analyses of change-from-baseline effect sizes across all study parameters in ERT-experienced patients demonstrated nominally significant improvements for patients switching from alglucosidase alfa to cipaglucosidase alfa plus miglustat across most study parameters. In contrast, patients remaining on alglucosidase alfa generally showed no change or worsening (56).

Taken together, clinical data demonstrate that the unique mechanism of action of cipaglucosidase alfa plus miglustat results in early reductions in glycogen levels (using Hex4 as a surrogate) and muscle damage (using CK as a surrogate). This leads to improvement in measures of muscle strength (MMT), motor function (6MWD and GSGC), and pulmonary function (FVC) (Figure 13). These benefits are maintained or progressively improve over time, as demonstrated in the open-label extension study for PROPEL (NCT04138277), in which a further 52 weeks of treatment (104 weeks in total) yielded durable improvements in biomarker levels and measures of muscle function compared with baseline. Importantly, patients who switched from alglucosidase alfa to cipaglucosidase alfa at week 52 also saw stabilization of or improvement in motor and respiratory function and biomarker levels over the subsequent 52 weeks of the extension period, supporting the robustness of the clinical responses attained with cipaglucosidase alfa plus miglustat (57). Similar durability of treatment effect was observed in another long-term study of cipaglucosidase alfa plus miglustat, wherein benefits on motor and respiratory function and biomarker levels were maintained for up to 48 months of follow-up in both ERT-experienced and ERT-naïve cohorts (46).

## 6 Discussion

**Figure 14:**
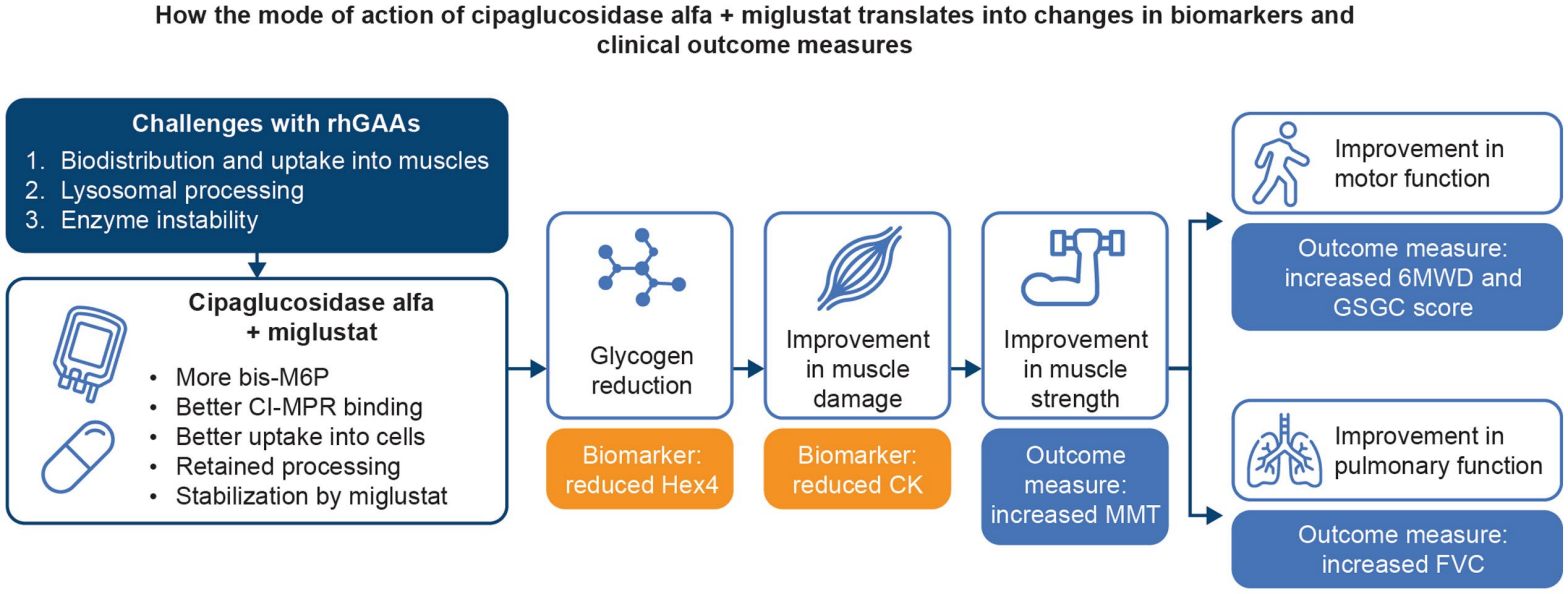
Summary of how the mechanism of action of cipaglucosidase alfa plus miglustat addresses the key challenges with Pompe ERTs and translates into improved outcomes for patients.

An extensive collection of *in vitro*, preclinical, and clinical data supports the hypothesis that treatment with cipaglucosidase alfa plus miglustat addresses the three key challenges of ERT for Pompe disease. By combining a bis-M6P-enriched enzyme that retains the capacity for complete processing with an enzyme stabilizer designed to minimize rhGAA inactivation during delivery, the combination therapy offers distinct advantages over existing ERTs, ultimately leading to improved outcomes for individuals living with LOPD (Figure 14).

Several therapeutic modalities are under investigation for Pompe disease, including substrate reduction therapy (58) and gene therapy (59–61), but ERT is the only approved therapy. Although clinical benefits have been attained in patients treated with alglucosidase alfa, the limited capacity for CI-MPR-mediated cellular uptake likely leads to declining efficacy over the long term (i.e., 3–5 years) (10–13), illustrating the need for more efficacious therapies. One newer ERT, avalglucosidase alfa, was developed to address the declining efficacy of ERT by using oxime chemistry to conjugate synthetic bis-M6P glycans onto rhGAA *N*-glycans to improve affinity for the CI-MPR (62). In a Phase III randomized trial (NCT02782741), treatment with avalglucosidase alfa led to improvements in some clinical outcomes compared with alglucosidase alfa in ERT-naïve patients (63), suggesting that addressing one of the limitations of rhGAA ERT (i.e., cellular uptake) can lead to beneficial downstream effects.

Cipaglucosidase alfa is the latest rhGAA to gain regulatory approval for LOPD and was developed with a different approach. Rather than using oxime chemistry to conjugate synthetic bis-M6P to rhGAA *N*-glycans, cipaglucosidase alfa is naturally produced from CHO cells selected for optimal rhGAA *N*-glycan profiles that include high levels of bis-M6P (25). Several lines of evidence support a benefit for naturally produced rhGAA versus rhGAA with synthetically modified glycans (30, 31). While synthetic bis-M6P can increase receptor binding, synthetic glycan conjugation also impairs the GAA processing that is required to attain optimal enzyme activity towards its natural substrate, glycogen, likely because of steric hindrance from bulky glycans near the active site of the enzyme (31). Furthermore, for naturally produced rhGAAs, *N*-glycans are removed following administration (in the endolysosomal pathway), resulting in a mature enzyme with a four- to 10-fold improvement in affinity and optimal enzyme activity for hydrolyzing glycogen (29, 30). This critically important GAA maturation process is blocked by incorporation of synthetic oxime bonds, resulting in an impairment of catalytic efficiency (30). Preservation of normal post-delivery processing is thus an important advantage for cipaglucosidase alfa as efficient lysosomal kinetics maximize the potential for glycogen clearance.

In addition to the benefits imparted by increased bis-M6P and retained *N*-glycan processing, the combination therapy of cipaglucosidase alfa plus miglustat also addresses the third limitation of ERT for LOPD by stabilizing the enzyme while in circulation, preventing inactivation. In mice, non-human primates, and adult individuals living with Pompe disease, miglustat increases plasma exposure (AUC) of cipaglucosidase alfa (25, 47). In *Gaa* −/− mice, this increased exposure, while lower than that observed in humans, results in higher clearance of skeletal muscle glycogen and greater increases in muscle strength for cipaglucosidase alfa plus miglustat versus cipaglucosidase alfa alone, supporting a direct benefit for target-cell pathology. Notably, cipaglucosidase alfa plus miglustat is the first approved ERT to utilize a small-molecule stabilizer to address the challenge of enzyme stability during and immediately after administration (64).

Overall, the novel mechanism of action of cipaglucosidase alfa plus miglustat has led to significant improvements in clinical responses in adults with LOPD, both in the Phase III PROPEL trial and throughout an ongoing extension trial for at least 2 years of total follow-up, in which analyses have demonstrated prolonged stability or improvement in key efficacy and safety outcomes both in patients treated with cipaglucosidase alfa plus miglustat and in those switched at 52 weeks from alglucosidase alfa to cipaglucosidase alfa plus miglustat (57). Beneficial treatment effects were also maintained in another long-term study, wherein patients treated with cipaglucosidase alfa plus miglustat experienced durable stabilization or improvement of motor and respiratory function and biomarker levels for up to 48 months of follow-up (46). This is in contrast to the 97-week follow-up data for avalglucosidase alfa, which revealed late declines in nearly every functional endurance and motor function outcome (e.g., upper- and lower-extremity handheld dynamometry, 6MWD, quick motor function test) despite earlier improvements upon initiation of treatment (65). While the extent to which these disparate long-term results are due to structural glycan differences, disparities in capacity for processing, or the presence/absence of a small-molecule stabilizer is unclear, the durability of the positive clinical outcomes for cipaglucosidase alfa plus miglustat supports the notion that the improved mechanism of action leads to tangible clinical benefits.

In the absence of head-to-head trials comparing avalglucosidase alfa with cipaglucosidase alfa plus miglustat, indirect treatment comparisons have been conducted, with conflicting results (66, 67). More clinical experience and real-world evidence will be necessary to fully demonstrate the strengths and limitations of each Pompe ERT. Nevertheless, a large body of *in vitro*, preclinical, and clinical evidence supports the hypothesis that treatment with a naturally bis-M6P-enriched rhGAA in combination with an enzyme stabilizer (i.e., cipaglucosidase alfa plus miglustat) can effectively address the three key challenges faced when treating Pompe disease with ERT.

## Data Availability

The datasets presented in this article are are available upon reasonable request. Requests to access the datasets should be directed to Nicholas A. Rees at nrees@amicusrx.com. Data sharing requests will be reviewed on a case-by-case basis.
