# Corrigendum: Cipaglucosidase alfa plus miglustat: linking mechanism of action to clinical outcomes in late-onset Pompe disease

**DOI:** 10.3389/fneur.2024.1540452

**Published:** 2025-01-03

**Authors:** Barry J. Byrne, Giancarlo Parenti, Benedikt Schoser, Ans T. van der Ploeg, Hung Do, Brian Fox, Mitchell Goldman, Franklin K. Johnson, Jia Kang, Nickita Mehta, John Mondick, M. Osman Sheikh, Sheela Sitaraman Das, Steven Tuske, Jon Brudvig, Jill M. Weimer, Tahseen Mozaffar

**Affiliations:** ^1^Department of Pediatrics in the College of Medicine, University of Florida, Gainesville, FL, United States; ^2^Metabolic Unit, Department of Translational Medical Sciences, University of Naples Federico II, Naples, Italy; ^3^Telethon Institute of Genetics and Medicine, Pozzuoli, Italy; ^4^Friedrich-Baur-Institute, Department of Neurology, LMU University Hospital, LMU Munich, Munich, Germany; ^5^Erasmus MC University Medical Center, Rotterdam, Netherlands; ^6^M6P-Therapeutics, St Louis, MO, United States; ^7^Amicus Therapeutics, Inc., Princeton, NJ, United States; ^8^Metrum Research Group, Tariffville, CT, United States; ^9^Incyte Corporation, Wilmington, DE, United States; ^10^Department of Neurology, University of California, Irvine, Irvine, CA, United States

**Keywords:** Pompe disease, glycogen storage disease type II, lysosomal storage disorders, enzyme replacement therapy, *n*-butyldeoxynojirimycin

In the published article, there was an error in Figure 13 as published. The error relates to the positive/negative value of some of the numbers on the axes for the lower MMT score and the GSGC total score in Figure 13A. For both lower MMT score and GSGC total score, the axis scale incorrectly read −3, −2, 1, 0, −1, −2, −3 from left to right, whereas the axis scale for lower MMT score should have been −3, −2, −1, 0, 1, 2, 3 and the axis scale for GSGC total score should have been 3, 2, 1, 0, −1, −2, −3. The corrected Figure 13 and its caption appear below.

**Figure 13:**
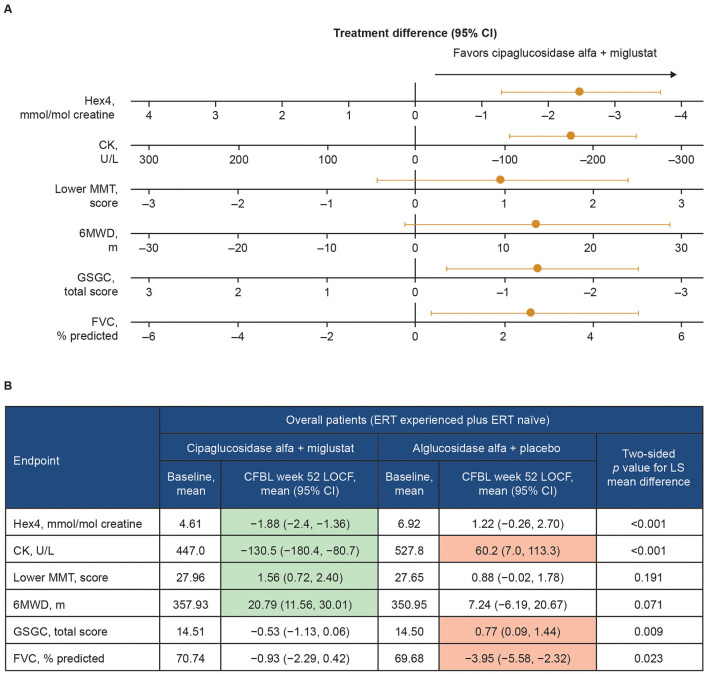
Change from baseline at week 52 of PROPEL—effect of cipaglucosidase alfa plus miglustat compared with alglucosidase alfa plus placebo in key efficacy outcomes. **(A)** Forest plot illustrating mean estimated treatment differences between cipaglucosidase alfa plus miglustat versus alglucosidase alfa plus placebo and corresponding 95% CIs are shown for the combined PROPEL study population for each outcome, with units as indicated on the x-axes. For all outcomes, right-sided directionality of treatment differences indicates favorable outcomes for cipaglucosidase alfa plus miglustat compared with alglucosidase alfa plus placebo. **(B)** The table shows baseline mean values and Week 52 CFBL values for cipaglucosidase alfa plus miglustat and alglucosidase alfa plus placebo. Shaded CFBL indicates nominally significant improvement (green) or nominally significant worsening (red) from baseline (i.e., the 95% CI does not include zero) within each treatment group. The *p*-values (two-tailed LS mean difference) shown in the far-right column are for the between-group treatment differences illustrated in the forest plot.

The authors apologize for this error and state that this does not change the scientific conclusions of the article in any way. The original article has been updated.

