# Supplementary material for: Cipaglucosidase alfa plus miglustat: linking mechanism of action to clinical outcomes in late-onset Pompe disease
Source: Front Neurol. 2024 Oct 18;15:1451512. doi: 10.3389/fneur.2024.1451512 (PMC11527667; doi:10.3389/fneur.2024.1451512)
Supplement: Supplementary file 1 [file Data_Sheet_1.PDF]

Supplementary Material

1 Methods

1.1 Column chromatography

Samples were subjected to cation-independent mannose 6-phosphate receptor (CI-MPR)–agarose affinity chromatography as previously described (1 mL of resin at a flow rate of 18 mL/h) by loading 1 µg of protein onto the column in 100 µL of column buffer (50 mM imidazole, 150 mM sodium chloride, 2 mM ethylenediaminetetraacetic acid [EDTA], 5 mM glycerophosphate, 0.05% v/v Triton X-100, 0.02% w/v sodium azide, pH 7.0) (1). The sample loop and column were washed with 4.5 mL of column buffer and eluted with a mannose 6-phosphate (M6P) gradient initially developed to 1 mM over 4.5 mL, then stepped to 5 mM M6P and held at 5 mM M6P for 3 mL to elute all bound material. Fractions (0.2 mL) were collected starting at the beginning of sample application in a 96-well plate and assayed for enzymatic activity. For determining the relative amount of acid α-glucosidase (GAA) in fractions, 25 µL of each fraction was transferred to a new 96-well microtiter plate, 25 µL of 1 mM 4-methylumbelliferyl α-D-glucopyranoside (4-MU-α-glc) in 100 mM sodium acetate at pH 4.8 was added, and the reaction was incubated at 37°C for 1 hour and stopped with 1.0 M glycine at pH 10.5. Fluorescence was measured using the Spectramax M2 (Molecular Devices, Sunnyvale, CA, USA) at 370 nm excitation, 460 nm emission. The Softmax Pro software (Molecular Devices, Sunnyvale, CA, USA) was used to convert the relative fluorescence units to nanomoles of 4-MU released per milliliter per hour (nmol/mL/h) by interpolation of a 4-MU standard curve.

1.2 CI-MPR plate binding assay

A 96-well plate binding assay was used to measure the maximum binding (B_{max}) of recombinant human (rh)GAA to the CI-MPR and determine the dissociation constant, K_D (defined as 0.5 B_{max}). Briefly, 50 µL of purified, soluble bovine CI-MPR (6 µg/mL) was applied to coat each of the 96 wells of a high-binding polystyrene enzyme immunoassay (Costar 3601) plate. After incubation for 1 hour, unbound CI-MPR was removed by washing the plate three times with 200 µL phosphate-buffered saline (PBS). A volume of 200 µL of 2% bovine serum albumin in PBS was added to each well to block non-specific binding. After blocking, the plate was washed three times with 200 µL of stripping buffer (100 mM sodium acetate, pH 4.0, 1 M NaCl, 0.02% v/v Tween 20) to remove proteins bound non-specifically. Each well was then washed three times with binding buffer (40 mm 2-[4-(2-hydroxyethyl)piperazin-1-yl]ethanesulfonic acid [HEPES], pH 6.5, 150 mM NaCl, 10 mM EDTA, 0.05% Tween 20). A concentration of 10 µg/mL of cipaglucosidase alfa or 200 µg/mL of alglucosidase alfa was added to a well and serially diluted three-fold to achieve an appropriate range of concentrations suitable for detection as bound to CI-MPR by measuring rhGAA enzyme activity. After 1 hour, unbound rhGAA was removed by washing each well three times with binding buffer (40 mm HEPES, pH 6.5, 150 mM NaCl, 10 mM EDTA, 0.05% Tween 20). Bound rhGAA was measured by enzyme activity based on the hydrolysis of a fluorogenic substrate, 4-MU-α-Glc. The amount of hydrolyzed 4-MU-α-Glc converted to 4-MU and glucose, determined by interpolation of nanomoles of 4-MU from a standard curve and rhGAA activity in units of nmol of 4-MU/mL/hour, was plotted against rhGAA concentration. The parameters B_{max} and K_D were determined from the non-linear regression curve fitted to the data using a one-site saturable binding model in GraphPad Prism (version 9.5.0).

1.3 Pompe patient fibroblast uptake assay

Pompe patient fibroblasts (25,000 cells/well) in 200 µL Dulbecco's Modified Eagle Medium high glucose + 10% fetal bovine serum were seeded in sterile, clear-bottom, 96-well black plates (Corning Costar Corp., Corning, NY, USA) and incubated at 37ºC, 5% CO_2 overnight. The cells were incubated with increasing concentrations (1.9 to 500 nM) of either cipaglucosidase alfa or alglucosidase alfa at 37ºC, 5% CO_2 for 16–18 hours. To be sure that the GAA activity measured in the cell lysate was only from internalized enzyme, 1.5 M Tris-HCl (pH >10) was added to the cells, and the mixture was incubated at room temperature for 30 minutes to inactivate any enzyme on the cell surface then neutralized with 1.0 M sodium phosphate. After washing the cells with Dulbecco's PBS, the cells were lysed in 50 µL of 0.25% Triton X-100 in water for 1 hour at 37°C. The lysate was divided for GAA enzyme activity assay and determination of protein concentration. For the enzyme assay, 20 µL of cell lysate was transferred to a clear-bottom, 96-well black plate (Corning Costar Corp., Corning, NY, USA; catalog # 3631), and 50 µL of 1 mM 4-MU-α-Glc in 100 mM sodium acetate, pH 4.8, was added. The reaction was incubated at 37°C for 1 hour and stopped with addition of 0.5 N sodium hydroxide. Fluorescence was measured with a Spectramax M2 (Molecular Devices, Sunnyvale, CA, USA) at 370 nm excitation, 460 nm emission. A 4-MU standard curve was used to interpolate the raw fluorescence units to nanomoles of 4-MU released per milliliter per hour (nmol/mL/h) with the Softmax Pro software. Protein concentration was determined by the Pierce Micro bicinchoninic acid (BCA) assay (ThermoFisher Scientific, Waltham, MA, USA). Briefly, 20 µL of cell lysate was transferred to a clear 96-well plate and total protein determined according to the assay instructions. To normalize for differences in cell density, enzyme activity (nmol 4-MU released/mL lysate/h) was divided by total lysate protein concentration (mg/mL) to determine specific activity (nmol 4-MU released/mg protein/h). The specific activity was used to plot uptake saturation curves in Prism (GraphPad Software, Inc., La Jolla, CA, USA). Data were analyzed by non-linear regression with data fitted to a rectangular hyperbola in GraphPad Prism to calculate the maximal uptake and the concentration of rhGAA that yielded half-maximal enzyme internalization (K_{uptake}).

1.4 Proteolytic processing of cipaglucosidase alfa *in cellulo*

Cell-uptake assays were performed as previously described with modified dose and timing (2). Briefly, fibroblasts from a patient with Pompe disease (Corielle Institute No. GM12932) were treated with 20 nM cipaglucosidase alfa for 18 hours of enzyme uptake, followed by washout and collection at various time points to allow for rhGAA processing. Harvested cells were processed for anti-GAA and anti-actin Western blot analysis (10 ng precursor enzymes and 3 µg lysates).

1.5 Phase I cipaglucosidase alfa plus miglustat dose modeling

Non-clinical studies were performed in male *Gaa* knockout (KO) mice to assess the effect of miglustat exposure on GAA activity in the blood and quadriceps and the resulting effect on glycogen reduction. Concentration–response relationships were explored to determine an optimal miglustat dose that would be expected to bind and stabilize rhGAA (cipaglucosidase alfa) while minimizing miglustat exposure in the quadriceps to prevent inhibition of GAA activity. Miglustat oral doses (1, 3, 5, 10, 20, and 30 mg/kg) were administered with 20 mg/kg intravenous doses of rhGAA in Pompe mice, and GAA activity was measured in plasma and the quadriceps (4-MU-Glc hydrolysis assay), along with glycogen levels. The concentration–response relationship was evaluated by examining the effect of the observed glycogen reduction (described as a function of the sum of effect for miglustat and rhGAA vs. rhGAA

alone) on the ratio of miglustat/rhGAA exposure (described as a ratio of average concentrations in µg/mL).

Results showed that co-administration of a 10 mg/kg dose of miglustat with a 20 mg/kg dose of rhGAA optimized the stability of GAA activity in plasma and maximized glycogen reduction in the quadriceps. Lower doses of miglustat (1, 3, or 5 mg/kg) resulted in suboptimal stabilization of GAA, whereas the highest dose of miglustat (30 mg/kg) likely resulted in excessive exposure to miglustat at the site of effect (quadriceps), as evidenced by lower glycogen reduction. The observed miglustat/rhGAA area under the concentration–time curve (AUC) ratio of 0.01159 (i.e., 10 mg/kg) corresponds to a miglustat dose of approximately 260 mg in a typical 70 kg human based on the predicted average concentrations of miglustat and rhGAA in humans (as determined with a non-clinical pharmacokinetic [PK] model).

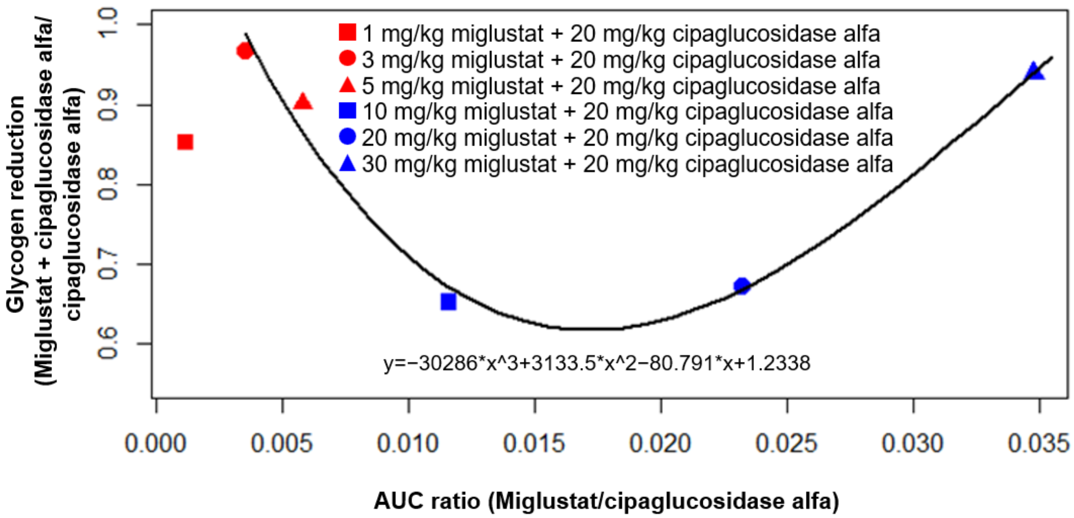

Miglustat dosing rationale was also explored with *in vitro* experiments examining the inhibitory effect of miglustat on GAA activity (rhGAA inhibition assay). The concentrations of miglustat associated with 50% of the maximum inhibitory effect (IC_{50}) on rhGAA activity at pH 7.0 (plasma) and pH 5.2 (lysosome) were 170 and 377 µg/L, respectively. Based on this information, concentration–time profiles of miglustat were simulated to determine the time above the IC_{50} (i.e., the duration over which GAA activity is expected to be inhibited by at least 50%) for pH values associated with plasma and lysosomal compartments. Simulations were also performed to determine the time above the IC_{50} in plasma and lysosome across various dose levels of miglustat. As shown in the table below, a 260 mg miglustat dose provided a maximal binding time above the IC_{50} in plasma of 17.5–17.9 hours, concurrent with a brief period of time above the IC_{50} in lysosomes of 3.58–4.15 hours.

Miglustat, mg	Time >IC_{50}, h	
	Plasma	Lysosome
100	13.5	0
150	15.0	0
200	16.4	1.19
233	17.2	2.96
**250**	**17.5**	**3.58**
**270**	**17.9**	**4.15**
300	18.4	4.92
466	20.7	8.04
600	22.0	9.96
699	22.8	11.2

1.6 Total plasma GAA protein profiles

Blood samples for plasma total GAA protein and plasma miglustat were taken at day 1 and week 52, just prior to the start of cipaglucosidase alfa infusion (time 0) and at 1, 4, 6, 12, and 24 hours after the start of infusion. Total GAA protein was measured through signature peptides unique to human GAA with a validated liquid chromatography–tandem mass spectrometry assay. A population PK model was prepared from pooled data in enzyme replacement therapy (ERT)-experienced and ERT-naïve adults with late-onset Pompe disease enrolled in the PROPEL (ATB200-03; NCT03729362) and ATB200-02 (NCT02675465) clinical trials. Separate population PK analyses (cipaglucosidase alfa/miglustat and alglucosidase alfa/placebo) were performed on the PROPEL study to assess differences between treatments.

1.7 Glycogen measurement in tissues (3)

Tissue lysates were prepared by homogenization of ~50 mg tissue for 5–10 seconds on ice with a micro-homogenizer (Pro Scientific, Thorofare, NJ, USA) in 500 µL deionized H_2O. An aliquot of each lysate was denatured at 100°C for 10 minutes to deactivate any endogenous glucosidase activity, centrifuged at 13,000 × *g* for 10 minutes at 4°C, and the supernatants retained. For the glycogen assay, 40 µL of diluted supernatants (1:8 dilution in H_2O for *Gaa* KO mouse samples and 1:4 dilution for wild-type [WT] mouse samples) and blanks of 40 µL of H_2O were added to 10 µL of amyloglucosidase (800 U/mL in 0.5 M sodium acetate, made fresh at the time of assay; Sigma #101155G-F) in a low-evaporation, transparent 96-well plate ('amylo-plus'). Furthermore, samples and blanks without amyloglucosidase digestion were created by assembling 40 µL of supernatants and 10 µL of 0.5 M sodium acetate ('amylo-minus'). All samples were analyzed in duplicate. In parallel, a glycogen standard curve of 3.125–400 µg/mL was prepared. All sample and glycogen standard plates were then incubated at 50°C for 1 hour, followed by denaturing at 100°C for 10 minutes and cooling to 4°C. A volume of 200 µL of glucose assay reagent (Sigma-Aldrich #G3293-50ML) was added to each well of standards and lysates. Plates were incubated at room temperature for at least 15 minutes and read with SpectraMax M2e for absorbance at 340 nm. For data analysis, readouts for 'amylo-plus' and 'amylo-minus' were subtracted by their respective blanks, then for each sample, the 'amylo-plus' absorbance

above blank was subtracted by its 'amylo-minus' signal. The resulting value was compared against the glycogen standard curve to interpolate the glycogen concentration of this sample. A BCA protein assay (Pierce, Rockford, IL, USA) was conducted according to the manufacturer's instructions to determine the total protein concentration in the non-denatured tissue lysates. The absolute glycogen concentration in lysates was normalized by the protein concentration, and the final glycogen level was expressed as microgram of glycogen per milligram of total protein (μg/mg protein).

1.8 Immunohistochemistry of muscle sections (1)

WT and Pompe *Gaa* KO mouse quadriceps tissue samples were fixed for 3 days at room temperature in Z-Fix (Anatech, Battle Creek, MI, USA). After two rinses in deionized water for 10 minutes each, samples were processed in a Tissue-Tek® VIP™ 5 vacuum infiltration processor (Sakura Finetek USA, Torrance, CA, USA) and embedded in paraffin with a Tissue-Tek® TEC™ 5 tissue embedding console system (Sakura Finetek). Transverse paraffin sections of 5 mm thickness were cut on an HM325 microtome (Microm, Dreieich, Germany) and mounted onto a SuperFrost Plus slide (ThermoFisher, Pittsburgh, PA, USA). Sections were dewaxed in xylene and rehydrated in deionized water. After heat-induced epitope retrieval in a decloaking chamber (Biocare Medical, Concord, CA, USA), the endogenous peroxidase activity was quenched by immersing the sections in 3% H_2O_2/Tris-buffered saline (TBS) solution, and the non-specific background was blocked by incubating the sections in 10% normal goat serum (Jackson ImmunoResearch, West Grove, PA, USA) prepared in TBS with 0.1% Tween 20. Sections were then incubated with a rabbit anti-lysosomal-associated membrane protein 1 (LAMP1) polyclonal antibody (Abcam, Cambridge, MA, USA) or a rabbit anti-LC3A monoclonal antibody (Cell Signaling Technology, Danvers, MA, USA) overnight at 4°C, followed by incubation with rabbit-on-rodent horseradish peroxidase polymer (Biocare Medical). The target of interest was visualized with a Betazoid DAB chromogen kit (Biocare Medical). Sections were counterstained with hematoxylin and mounted in Acrytol mounting media.

1.9 PK/pharmacodynamic analyses of change from baseline

Serum hexose tetrasaccharide and creatine kinase measurements were performed as previously described (4). Change-from-baseline calculations for key efficacy outcomes were performed using an analysis of covariance model with last observation carried forward.

1.10 Functional muscle strength tests

Muscle-function assessments (wire-hang and grip-strength tests) were performed once a month for 5 months, 7 days after drug administrations, as previously described (1). Briefly, for the wire-hang assay, mice were placed in an inverted grid approximately 60 cm above a cage containing a soft cushion of bedding. The maximum duration that the mice were able to hang onto the grid (latency of fall) was measured with a timer. If a mouse did not fall after 2 minutes, a value of 2 minutes was recorded. This procedure consisted of one trial per mouse on two independent days, with the average value of the latency to fall reported. For the grip-strength assay, the test was conducted with an axial force transducer grip meter (purchased from Columbus Instruments International Corporation, Columbus, OH, USA), which is specifically designed and recommended for measuring mouse grip strength. The meter was set to zero before starting, and grams as a unit/scale of value was chosen. With the grip-strength meter set on a vertical stable surface, the forepaws of each mouse were placed on a triangle bar 'trapeze', triggering a gripping response on the bar by the mouse. Care was taken to visually ensure that both front limbs of the mouse were gripping the bar. The animal was pulled in one fluid motion away from the grip meter. The strength with which it gripped the trapeze while being pulled was

recorded. This procedure consisted of three consecutive trials for each animal in 1 day, and the average of the three trials (obvious outliers were masked and excluded from the average) was reported.

References

1. Xu S, Lun Y, Frascella M, Garcia A, Soska R, Nair A, et al. Improved efficacy of a next-generation ERT in murine Pompe disease. *JCI Insight* (2019) 4. doi: 10.1172/jci.insight.125358

2. Selvan N, Mehta N, Venkateswaran S, Brignol N, Graziano M, Sheikh MO, et al. Endolysosomal N-glycan processing is critical to attain the most active form of the enzyme acid alpha-glucosidase. *J Biol Chem* (2021) 296:100769. doi: 10.1016/j.jbc.2021.100769

3. Gotschall R, Xu S, Lun Y, Schilling A, Soska R, Frascella M, et al. Novel recombinant human acid α-glucosidase with optimal glycosylation is significantly better than standard of care enzyme replacement for glycogen clearance in skeletal muscles of GAA knock-out mice. *Mol Genet Metab* (2015) 114:S49. doi: 10.1016/j.ymgme.2014.12.096

4. Schoser B, Roberts M, Byrne BJ, Sitaraman S, Jiang H, Laforet P, et al. Safety and efficacy of cipaglucosidase alfa plus miglustat versus alglucosidase alfa plus placebo in late-onset Pompe disease (PROPEL): an international, randomised, double-blind, parallel-group, Phase 3 trial. *Lancet Neurol* (2021) 20:1027-37. doi: 10.1016/S1474-4422(21)00331-8

frontiers
